# The association of maternal and infant early gut microbiota with respiratory infections in infants

**DOI:** 10.1038/s41390-025-04326-0

**Published:** 2025-08-20

**Authors:** Sanni Hyvönen, Aki Saarikivi, Jani Mälkönen, Terhi Solasaari, Katri Korpela, Willem M. de Vos, Anne Salonen, Terhi Ruuska-Loewald, Kaija-Leena Kolho

**Affiliations:** 1 Department of Pediatrics, Tampere University Hospital, Tampere, Finland; 2 Faculty of Medicine, University of Helsinki, Helsinki, Finland; 3 Helsinki University Hospital, Helsinki, Finland; 4 Children’s Hospital HUS, University of Helsinki, Helsinki, Finland; 5 Pediatric Clinic, Social Services and Health Care Division, City of Helsinki, Helsinki, Finland; 6 Human Microbiome Research Program, Faculty of Medicine, University of Helsinki, Helsinki, Finland; 7 Laboratory of Microbiology, Wageningen University, Wageningen, The Netherlands; 8 Research Unit of Clinical Medicine, University of Oulu, Oulu, Finland; 9 Department of Pediatrics and Adolescent Medicine, Oulu University Hospital, Oulu, Finland; 10 Biocenter Oulu, University of Oulu, Oulu, Finland

## Abstract

**Background:**

There are limited data on the proposed association of early gut microbiota composition and the risk of respiratory tract infections (RTI) in infants from prospective studies.

**Methods:**

We investigated the maternal and infant gut microbiota in infants prospectively followed up for their RTIs in the HELMi cohort from Helsinki, Finland. The 16S rRNA gene amplicon data was assessed at weeks 3 and 6 from 461 infants, of whom 178 developed RTIs within 3 and 6 months of life. Fecal samples collected near the due date were available from 261 mothers.

**Results:**

There was no difference in the maternal or early infant gut microbiota in the overall microbiota composition in alpha or beta diversity between infants with or without RTIs within the first 3 and 6 months of life. The relative abundances of adult-type butyrate producers and some *Enterobacteriaceae* were significantly more higher at 3 and to some extent also at 6 weeks of age in the infection group compared to controls, while their mothers’ microbiota was significantly enriched with *Enterococcus, Citrobacter, and Enterobacter* spp., and *Clostridium* being less abundant.

**Conclusion:**

The maternal and early-life infant gut microbiota may play a role in predisposition to RTIs in infants.

**Impact:**

- The maternal and early-life infant gut microbiota profile was associated with infants’ respiratory tract infections within the first 6 months of life.
- In infants, the higher abundance of adult-type butyrate producers and some *Enterobacteriaceae* were associated with respiratory tract infections, while mothers’ microbiota was significantly enriched with *Enterococcus, Citrobacter, and Enterobacter* spp. in the group of infants with infections.
- The results indicate that maternal and infant gut microbiota may play a role in predisposing an infant to infections during early life. Further studies are warranted on how this link is mediated.

## Introduction

Healthy, full-term infants experience four to ten respiratory tract infection (RTI) episodes in the first year of life in a high-income country.^1–3^ The full burden of RTIs is seen not only in the reduced quality of life in infants but also in the socioeconomic effects including parental worry and absenteeism from work.^4–10^

Early gut microbiota composition has been suggested to be associated with RTIs in children.^11,12^ There is evidence from animal studies that early-life gut microbiota composition might influence respiratory immunity and, therefore, increase the susceptibility to RTIs.^13,14^ However, results from human studies are inconsistent. There is evidence that low alpha diversity and low relative abundance of particular gut-commensal bacterial genera (*Bifidobacterium, Faecalibacterium, Ruminococcus, and Roseburia)* are associated with childhood respiratory diseases, especially wheezing, and asthma,^15–20^ whereas evidence between gut microbiota and RTIs in infancy is less studied due to a lack of longitudinal large studies with fecal sampling during the first months of life, standard follow-up times, and definitions of RTIs.^20–25^

Most birth cohort studies^15–18,26^ and case-control studies^19–21^ investigating the relationship between the gut microbiota and RTIs have explored wheezing or asthma. Only a few studies have reported RTIs as an outcome.^22–25^ Furthermore, there are limited data on the association between the gut microbiota and RTIs in infants using a careful follow-up of respiratory symptoms in prospective cohort studies.^22,24,25^ Finally, maternal microbiota has rarely been investigated in previous studies. Considering the suggested role of gut microbiota composition in the risk of childhood RTIs, we hypothesized that the early gut microbiota is associated with the occurrence of RTIs in the first 6 months of life.

In this prospective study cohort, using a systematic follow-up of respiratory symptoms, we set out to investigate the proposed association between early gut microbiota composition and the occurrence of RTIs in infants.

## Methods

### Study design

This was a nested case-control study retrieved from a prospective HELMI cohort study (see below the details of the cohort). We compared the maternal and infant gut microbiota between infants with an RTI episode within 3 and 6 months of life and infants with no such episodes serving as controls. Infants with an RTI episode were defined as infants who presented with a lower RTI (LRTI) or an upper RTI (URTI) episode with fever or otitis media in the first 6 months of life. In the HELMI cohort study, the families used a prospective online diary weekly for the first 4 months of life, then biweekly until 7 months, including infection symptoms and doctoral visits, which was to detect infants who developed RTIs in the first 6 months of life and their controls without such infections.^1^

The study population of mothers and infants originated from the prospective HELMi (Health and Early Life Microbiota) birth cohort recruited in the Helsinki region of Finland, from February 2016 to March 2018. A detailed description of the HELMi cohort has previously been published.^27^ In brief, the inclusion criteria were singleton, term newborn infants born healthy, with birth weight exceeding 2.5 kg. To study the gut microbiota composition, we used fecal samples from mothers collected close to the due date (±2 weeks) and infant samples collected at weeks 3 and 6. Parents collected the fecal samples at home, froze them immediately at −20 °C, and transported them in a frozen form to the laboratory, which kept them at −80 °C until DNA extraction. Parental background data were recorded at the enrollment.^27,28^

### Study groups

**Fig. 1:**
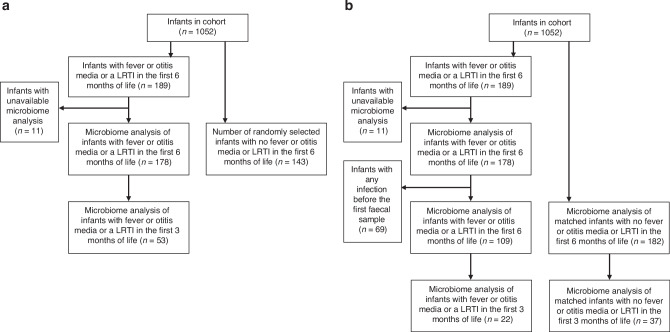
Selection of the study groups from the original HELMi Cohort. The flowchart shows the selection of study groups from the original cohort of 1052 infants. In analysis a, randomly selected controls were used, and in the sensitivity analyses (analysis b), matched controls were included.

From the original HELMi cohort of 1052 infants, 189 (18%) infants developed an RTI episode in the first 6 months of life (Fig. 1). Of 189 infants, there were good quality microbiota data available from 178 infants and 136 mothers (Table 1). A random sample of infants with no RTI episodes during 6 months of life (*n* = 143) and their mothers (*n* = 125) served as controls. Baseline characteristics were comparable between the groups except there were more first-born infants in the control group (Table 2). In total, we included samples from 461 infants and 261 mothers for microbiota analyses. In the initial analyses, we compared the 3- and 6-week microbiota between infants with RTI episodes in the first 6 months of life and the control infants. For the sensitivity analysis, after excluding infants with any RTIs before the first fecal sample was taken, we had samples of 109 infants who developed an RTI episode in the first 6 months of life and 182 controls (Fig. 1) matched for sex, year of birth, season of birth, mode of delivery, exposure to intrapartum antibiotics and the number of older siblings (Supplemental Table S1).

**Table 1:** Successful microbial analysis in different groups of the study population.

	No. of samples with successful microbial analysis	
Mothers of infants with RTI in the first 6 months of life	136	
Mothers of infants with no RTI in the first 6 months of life	125	
	No. of samples with successful microbial analysis taken at 3 weeks of age	No. of samples with successful microbial analysis taken at 6 weeks of age
Infants with RTI in the first 3 months of life	53	53
Infants with RTI in the first 6 months of life	178	177
No. of randomly selected infants with no RTI in the first 6 months of life	143	139
Infants with RTI in the first 3 months of life. Infections before 3-week fecal sample excluded.	22	
Matched infants with no RTI in the first 3 months of life. Infections before 3-week fecal sample excluded.	37	
Infants with RTI in the first 6 months of life. Infections before 3-week fecal sample excluded	109	
Matched infants with no RTI in the first 6 months of life. Infections before 3-week fecal sample excluded	182	

*RTI*, respiratory tract infection with fever or otitis media or a lower respiratory tract infection.

**Table 2:** Baseline Characteristics of Study Population.

	categorical: *n* (%) numeric: mean (SD)			
	Infants with RTI in the first 3 months (*n* = 53)	Infants with RTI in the first 6 months (*n* = 178)	Infants with no RTI (*n* = 143)	*p* value
Sex, male	32 (60.4%)	95 (53.4%)	70 (49.0%)	0.20^*^ 0.44^**^
Year of birth				
2016	22 (41.5%)	63 (35.4%)	65 (45.5%)	0.63^*^ 0.08^**^
2017	26 (49.1%)	105 (59.0%)	70 (49.0%)	1.00^*^ 0.09^**^
2018	5 (9.4%)	10 (5.6%)	8 (5.6%)	0.34^*^ 1.00^**^
Season of birth				
Winter	15 (28.3%)	28 (15.7%)	31 (21.7%)	0.35^*^ 0.19^**^
Spring	10 (18.9%)	36 (20.2%)	30 (21.0%)	0.84^*^ 0.89^**^
Summer	10 (18.9%)	65 (36.5%)	42 (29.4%)	0.15^*^ 0.19^**^
Autumn	18 (34.0%)	49 (27.5%)	40 (28.0%)	0.48^*^ 1.00^**^
Mode of delivery				
Cesarean delivery	8 (15.1%)	24 (13.5%)	21 (14.7%)	1.00^*^ 0.87^**^
- Mother received antibiotics	8 (100%)	24 (100%)	21 (100%)	1.00^*^ 1.00^**^
- Vaginal birth	45 (85.0%)	154 (86.5%)	122 (85.3%)	
- Mother received antibiotics	10 (22.2%)	36 (23.4%)	29 (20.3%)	0.60^*^ 1.00^**^
Number of siblings in household				
No siblings	11 (20.8%)	62 (34.8%)	79 (55.2%)	<0.001^*^ <0.001^**^
≥1 sibling	42 (79.2%)	116 (65.2%)	64 (44.8%)	
Breastfeeding	52 (98.1%)	174 (97.8%)	143 (100%)	0.27^*^ 0.13^**^
Exclusive (months)	3.2 (1.9 SD)	3.3 (1.9 SD)	3.5 (1.7 SD)	0.36^*^ 0.41^**^
Partial (months)	11.1 (2.3 SD)	10.7 (2.8 SD)	11.0 (2.4 SD)	0.90^*^ 0.32^**^
Any use of probiotics during pregnancy	39 (73.6%)	138 (77.5%)	95 (51.0%)	0.39^*^ 0.03^**^
Antibiotic use before 3-week fecal sample	5 (9.4%)	7 (3.9%)	1 (0.7%)	0.006^*^ 0.08^**^
Antibiotic use before 6-week fecal sample	17 (32%)	22 (12.4%)	2 (1.4%)	<0.001^*^ <0.001^**^
Antibiotic use in the first 6 months of life		33 (18.5%)	3 (2.1%)	< 0.001^**^
Vaccination at 12 months^a^				
Full program	36 (67.9%)	135 (75.8%)	110 (76.9%)	0.22^*^ 0.88^**^
Partial program	9 (17.0%)	24 (13.5%)	17 (11.9%)	0.34^*^ 0.74^**^
No vaccination	4 (7.5%)	8 (4.5%)	7 (4.9%)	0.49^*^ 1.00^**^
Furry pet at home	24 (45.3%)	59 (33.1%)	46 (31.7%)	0.10^*^ 0.91^**^
Mother’s age	32.8 (4.0 SD)	32.8 (4.0 SD)	32.5 (4.1 SD)	0.70^*^ 0.51^**^
Father’s age	34.2 (4.5 SD)	34.4 (4.8 SD)	34.8 (5.6 SD)	0.46^*^ 0.56^**^
Maternal education				
Secondary school/upper secondary/vocational school	10 (18.9%)	22 (12.4%)	12 (8.4%)	0.07^*^ 0.28^**^
University including polytechnic	43 (81.1%)	156 (87.6%)	131 (91.6%)	
Paternal education				
Secondary school/upper secondary/vocational school	13 (24.5%)	45 (25.3%)	42 (29.4%)	0.59^*^ 0.45^**^
University including polytechnic	40 (75.5%)	132 (74.2%)	99 (69.2%)	
Maternal smoking	0 (0.0%)	0 (0.0%)	0 (0.0%)	
Paternal smoking	3 (5.7%)	27 (15.2%)	20 (14.0%)	0.14^*^ 0.87^**^
At least one parent with asthma	7 (13.2%)	26 (14.6%)	12 (8.4%)	0.41^*^ 0.12^**^
Mother with asthma	7 (13.2%)	18 (10.1%)	8 (5.6%)	0.13^*^ 0.16^**^
Father with asthma	1 (1.9%)	9 (5.1%)	5 (3.5%)	0.99^*^ 0.59^**^
At least one parent with autoimmune disease	13 (24.5%)	25 (14.0%)	21 (14.7%)	0.14^*^ 0.87^**^
Mother with autoimmune disease	9 (17.0%)	17 (9.6%)	10 (7.0%)	0.05^*^ 0.55^**^
Thyroid disease	5 (9.4%)	11 (6.2%)	4 (2.8%)	
Celiac disease	2 (3.8%)	3 (1.7%)	5 (3.5%)	
Other^b^	2 (3.8%)	3 (1.7%)	2 (1.4%)	
Father with autoimmune disease	4 (7.5%)	9 (5.1%)	11 (7.7%)	0.99^*^ 0.36^**^
Inflammatory bowel disease	3 (5.7%)	6 (3.4%)	6 (4.1%)	
Celiac disease	0 (0.0%)	1 (0.6%)	3 (2.1%)	
Other^c^	1 (1.9%)	4 (2.2%)	4 (2.8%)	

RTI, respiratory tract infection with fever or otitis media or a lower respiratory tract infection.

Data missing (n): Vaccination (4-11-9), Father’s age (0-1-1), Paternal smoking (0-1-3) Paternal education (0-1-2).

Statistically significant comparisons (*p* < 0.05) are shown in bold.

^*^The comparison is between infants with RTI in the first 3 months of age and infants with no RTI.

^**^The comparison is between infants with RTI in the first 6 months of age and infants with no RTI.

^a^The national immunization program: rotavirus, pneumococcus, diphtheria-tetanus-pertussis-polio-Hib, measles-mumps-rubella, and varicella-zoster virus.

^b^Inflammatory bowel disease, Type 1 diabetes, Psoriasis, Rheumatic disease.

^c^Thyroid disease, Type 1 diabetes, Psoriasis, Rheumatic disease.

### Sample size

In the previous birth cohort studies investigating the relationship between gut microbiota and the occurrence of RTIs, the sample size has been around 120 children^22,24^ and in case-control studies, the included number of children in the microbiota analyses have ranged from 49 to 155 altogether.^21,23^ These studies have found statistically significant differences in alpha- and beta-diversity of gut microbiota.^21–24^ Thus, the sample size of the study was deemed sufficient.

### Ethics

The study protocol was reviewed by the ethical committee of The Hospital District of Helsinki and Uusimaa (263/13/03/03 2015), Finland. The study was conducted following the principles of the Helsinki Declaration. All families gave their written informed consent for the HELMi study.

### Microbiota and statistical analyses

The data on infections and background variables were presented as a median and interquartile range (IQR) unless otherwise stated. Fecal DNA extraction and preparation of the samples for V3-V4 16S ribosomal ribonucleic acid (rRNA) gene amplicon sequencing has been previously described.^28^ Samples with <2000 reads were excluded from the analyses. Statistical analyses were performed using the R package mare.^29^ Alpha diversity was calculated as the inverse Simpson diversity index and richness as the number of OTUs (operational taxonomic unit). For beta-diversity analyses, unsupervised Principal-coordinate Analyses (PcoA) were calculated with the capscale function and the Bray-Curtis dissimilarities with the function vegdist of the R package vegan, complemented with permutational ANOVA using adonis function of the same package.^30^

GroupTest function of the mare package was used for comparison of the relative abundances of bacterial genera and families between the groups. The function selects the most optimal model for each taxon based on its distribution, using either the glm.nb function from the MASS package,^31^ lm function from base R with log-transformation if necessary, or the gls function from the nlme package.^32^ The GroupTest function calculates a model that is appropriate for each taxon separately and attempts to find a suitable model for the taxon. Each model is checked for fulfilling the assumptions (residual heteroscedasticity and normality). If these are not met, the model is corrected to fulfill the criteria (e.g., by including a residual variance parameter in the model). This level of scrutiny is normally not done, but rather all taxa are tested using the same model, which is unlikely to fit all taxa equally due to differences in their distributions. Thus, the presented *p* values are more robust than what is typical in the field. When no suitable model is found, and the model assumptions are not met, no *P* value is reported for the taxa. The total read counts per sample were used as the offset in the models. Standard Benjamini-Hochberg corrections for false discovery rate (FDR) were applied. FDR-corrected *P* values of <0.1 were considered significant. This research paper is an exploratory analysis in early-stage research, which justifies FDR < 0.1 in this study.

After this, we conducted a sensitivity analysis, by excluding infants with any infections before the first fecal sample was taken. Gut microbiota compositions were compared between infants who developed an RTI episode and control infants matched for sex, year of birth, season of birth, mode of delivery, exposure to intrapartum antibiotics, and the number of older siblings (see below and Supplemental Table S1).

The main analysis was based on the assumption that gut microbiota composition may reflect the association of environmental and perinatal factors on the risk of RTIs. The rationale for the analyses using matched controls was that the selected covariates may act as confounding covariates as they influence both the gut microbiota composition and the risk of RTIs.

## Results

From the HELMI cohort, we included all 178 infants who developed an RTI episode within the first 6 months of life and had an available fecal sample at weeks 3 and 6 as well as 143 infants without such infections serving as controls, and their mothers (*n* = 261) (Table 2, Supplemental Table S1).

### RTI episodes

In the 178 infants with an RTI episode within the first 6 months of life, the median duration of RTI symptoms was 11 days (IQR 7–15) (Table 3). Most of these RTIs were URTIs, including fever 49%, followed by otitis media 47%, and LRTIs 4%. Of the 178 infants with an RTI episode, 30% (*n* = 53) developed at least one episode during the first 3 months of life and a median duration of 10 days (IQR 6.5-15). Of these early RTIs, otitis media was the most common (53%), followed by URTI with fever 41% and LRTI 6%. During the first 6 months of life, 61% (*n* = 108/178) of infants with an RTI had a visit to a doctor, and 13.5% (*n* = 24/178) visited the hospital emergency department. Among the control group, 21% (*n* = 30/143) reported a doctor’s visit (not listed as an RTI episode), and 2% (*n* = 3/143) visited the hospital emergency department without a specific diagnosis. None of the infants died.

**Table 3:** Number of RTIs in infants with analyzed stool samples and in 1052 full-term infants from the original cohort during their first 6 months of life.

	Infants with RTI *n* = 178	Infants with no RTI *n* = 143	Original cohort of 1052 infants
**Upper respiratory tract infection including only fever**			
Proportion of infants with episodes (No)			
0–3 months	12% (22)	0 (0%)	2.1% (22)
0–6 months	56% (99)	0 (0%)	10% (109)
Total no. of episodes			
0–3 months	22	0	22
0–6 months	109	0	119
**Otitis media**			
Proportion of infants with episodes (No)			
0–3 months	16% (28)	0 (0%)	2.7% (28)
0–6 months	49% (87)	0 (0%)	9.3% (98)
Total no. of episodes			
0–3 months	28	0	28
0–6 months	105	0	120
**Lower respiratory tract infection**			
Proportion of infants with episodes			
0–3 months	2.3% (3)	0% (0)	0.3% (3)
0–6 months	3.9% (7)	0% (0)	0.7% (7)
Total no. of episodes			
0–3 months	3	0	3
0–6 months	8	0	8

*RTI*, respiratory tract infection with fever or otitis media or a lower respiratory tract infection.

### Maternal gut microbiota composition and RTI episodes in infants

**Fig. 2:**
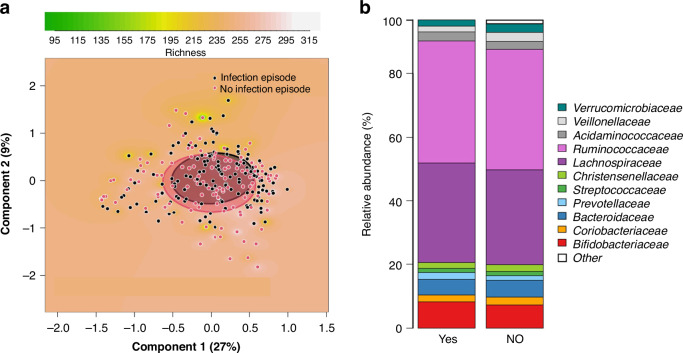
Principal coordinate analysis (PCoA) and relative abundances of the maternal microbiota at the family level. Comparison between mothers of infants who developed a respiratory tract infection episode (RTI) in the first 6 months of life and mothers of randomly selected infants with no such infection episode. PCoA plots based on Bray-Curtis dissimilarities of the samples, showing the richness of the microbiota as background (**a**). Clusters are shown by circles, which were drawn based on the standard deviations of the data points in each category of the samples (**a**). The comparisons are between mothers of infants who developed an RTI in the first 6 months of life and mothers of infants remaining healthy (*p* = 0.39). Clustered stacked column graphs demonstrate microbiota differences at the family level (**b**). The comparisons are between mothers of infants who developed an RTI in the first 6 months of life (YES) and mothers of infants remaining healthy (NO).

Based on the beta diversity of microbiota, we found no statistically significant differences in the gut microbiota composition between mothers of infants with and without an RTI episode during the first 6 months of life (Fig. 2). Beta diversity analysis, representing the dissimilarities between microbial communities using PCoA, showed no differences between the gut microbiota of mothers of infants with and without an RTI episode at family level (*p* = 0.39) (Fig. 2a) or at genus level (*p* = 0.30) (Supplemental Fig. S1a). The microbial richness and diversity defined by the number of OTUs did not differ between mothers of infants developing an RTI episode and mothers of controls (Supplemental Table S2).

In the taxonomic distribution, the most predominant bacteria of the maternal microbiota on family level were *Ruminococcaceae* (38% in the RTI group and 38% in the control group) and *Lachnospiraceae* (31% and 30%, respectively) (Fig. 2b). The relative abundance analysis at family level revealed that mothers of infants with RTI had a higher abundance of *Enterococcaceae* and *Enterobacteriaceae* together with *Family XIII Incertae Sedis* and *Pasteurellacceae*, while *Clostridiaceae* was less abundant compared to mothers of infants with no infections (Table 4). At genus level mothers of infants with RTI had a higher abundance of *Enterococcus*, *Enterobacter*, and *Citrobacter* and a lower abundance of *Clostridium* (Table 4). All microbiota results in the relative abundance analysis between mothers on family and genus levels in the microbiota samples are listed in Supplemental Tables S3 and S4.

**Table 4:** Maternal microbiota differences at family and genus level.

Family			Genus		
	p_FDR	Fold Change		p_FDR	Fold Change
**Taxon**					
Firmicutes_Bacilli_Lactobacillales_*Enterococcaceae*	1.59e-11	99.0	Firmicutes_Bacilli_Lactobacillales_*Enterococcaceae_Enterococcus*	3.42e-11	99.0
Proteobacteria_Gammaproteobacteria_Enterobacteriales_*Enterobacteriaceae*	1.08e-06	3.57	Proteobacteria_Gammaproteobacteria_Enterobacteriales_*Enterobacteriaceae_Enterobacter*	2.43e-06	3.51
Proteobacteria_Gammaproteobacteria_Pasteurellales_*Pasteurellaceae*	0.091	2.01	Proteobacteria_Gammaproteobacteria_Enterobacteriales_*Enterobacteriaceae_Citrobacter*	8.74e-05	3.29
Firmicutes_Clostridia_Clostridiales_*FamilyXIIIIncertaeSedis*	0.069	1.31	Firmicutes_Clostridia_Clostridiales_*Clostridiaceae_Clostridium*	0.067	0.55
Firmicutes_Clostridia_Clostridiales_*Clostridiaceae*	0.042	0.546			

FDR-corrected *P* values for microbiota differences between mothers of infants with a respiratory tract infection episode in the first 6 months of life and mothers of randomly selected infants remaining healthy in the first 6 months of life.

Standard Benjamini-Hochberg corrections for false discovery rate were applied.

*FDR* false discovery rate.

### Early gut microbiota composition and RTI episodes

**Fig. 3:**
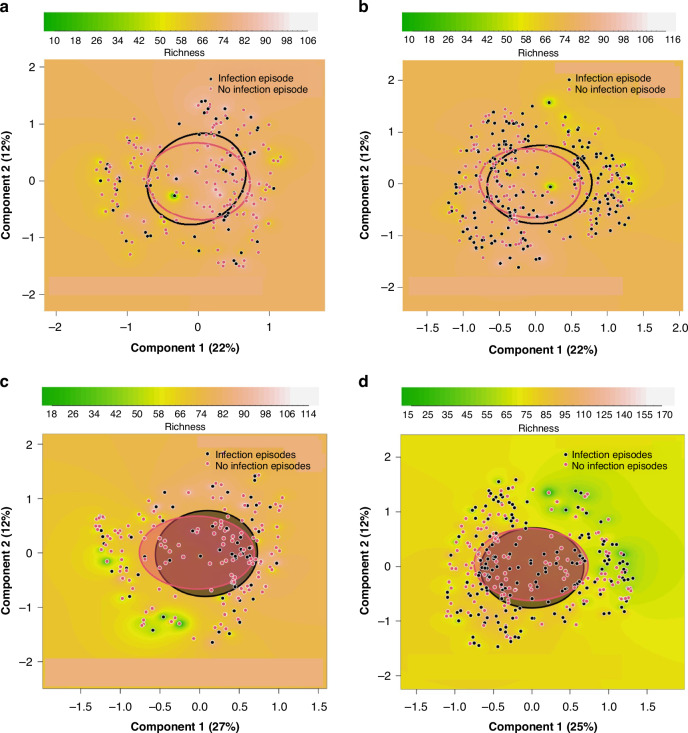
Principal coordinate analysis (PCoA) of the infant microbiota at the family level between infants who developed respiratory tract infection (RTI) episodes in the first 3 and 6 months of life and infants in the control group. PCoA plots are based on Bray-Curtis dissimilarities of the samples, and show richness as the background at time points of 3 (**a**, **b**) and 6 weeks of age (**c**, **d**). The comparisons are between infants who developed an RTI episode in the first 3 (**a**, *p* = 0.87 and **c**, *p* = 0.39) and 6 (**b**, *p* = 0.43 and **d**, *p* = 0.95, respectively) months of life and randomly selected infants remaining healthy in the first 6 months of life.

**Fig. 4:**
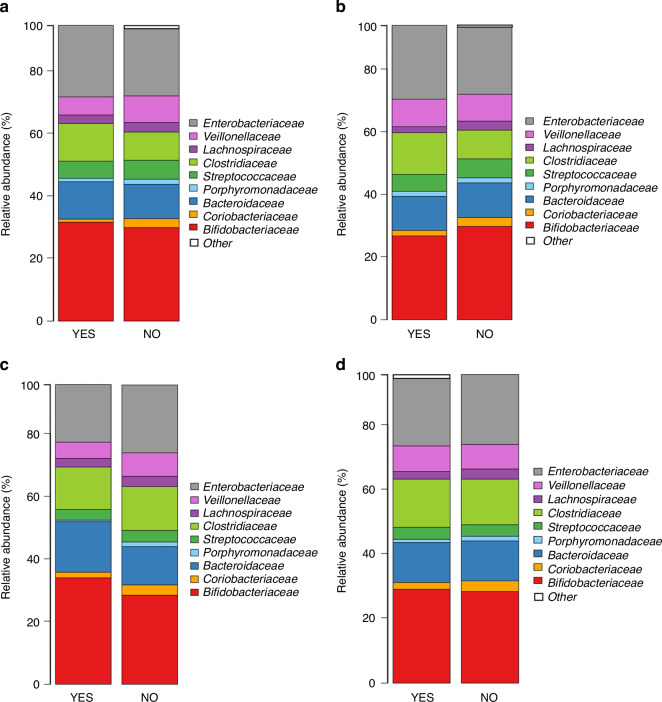
Relative abundances of the infant microbiota in infants who developed a respiratory tract infection episode (RTI) in the first 3 and 6 months of life and infants in the control group. Clustered stacked column graphs demonstrate microbiota differences at the family level at time points of 3 and 6 weeks of age. At week three and six, the comparisons are between infants who developed an RTI episode (YES) in the first 3 (**a**, **c**) and 6 (**b**, **d**) months of life and randomly selected infants remaining healthy (NO) in the first 6 months of life.

Based on beta diversity, there was no difference in the overall composition of microbiota at 3 or 6 weeks of age between infants who developed an RTI episode and the control group (Figs. 3–4). At 3 weeks of age, we observed no significant difference in PCoA between infants who developed an RTI episode within the first three (*p* = 0.87) or 6 months (*p* = 0.43) of life when compared to infants in the control group at microbiota family level (Fig. 3a, b) nor at genus level within the first three (*p* = 0.68) or 6 (*p* = 0.34) months of life (Supplemental Fig. S2a, b). We did not find significant differences at 6 weeks of age in PCoA between infants developing RTI episodes within the first 3 (*p* = 0.39) or 6 (*p* = 0.95) months of life when compared to infants in the control group at family level (Fig. 3c, d) nor at genus level within the first 3 (*p* = 0.46) or 6 (*p* = 0.93) months of life (Supplemental Fig. S2c, d). At weeks 3 and 6 the overall microbial richness and diversity defined by the number of OTUs showed no differences between infants developing an RTI episode during the first 3 or 6 months of life and the controls (Supplemental Table S2).

In the taxonomic distribution, the two most predominant bacteria of the microbiota at family level were *Bifidobacteriaceae* and *Enterobacteriaceae* at the age of 3 and 6 weeks in all infant groups (Fig. 4). The most significant difference in the microbiota composition at family level in the fecal samples at 3 weeks of age was that infants who developed an RTI within the first 3 months of life had a higher abundance of *Rikenellaceae* and *Verrucomicrobiaceae*, followed by increased *Prevotellacaeae, Actinomycetaceae, Coriobacteriaceae* and *Micrococcaceae* compared to that in control group (Table 5). The higher abundance of *Prevotellacaeae* was also seen in infants developing RTI within the first six months of life. Only this higher abundance of *Prevotellacaeae* persisted in the samples taken at 6 weeks of age but only in infants developing an RTI within the first 3 months of life (Table 6). At genus level, in the samples taken at 3 weeks of age, altogether 11 genera (*Alistipes* and *Akkermansia* were the most elevated followed by *Peptoniphilus, Faecalibacterium*, and *Serratia)* were increased in infants developing an RTI within the first 3 months of life compared to that in the control group (Table 5). The higher relative abundance of *Faecalibacterium*, a major butyrate producer, and *Peptoniphilus* was also observed in infants developing RTIs within the first 6 months of life, while *Anaerostipes* was less abundant (Table 5). The relative abundance analysis from the samples taken at 6 weeks of age revealed a concomitant strong decrease of *Anaerostipes* in infants developing an RTI within the first three and 6 months of life (Table 6). We also found a higher abundance of six genera (*Prevotella*, *Macellibacteroides, Catenibacterium, Subdoligranulum, Faecalibacterium*, and *Atopobium*) in infants developing infection within the first 3 months of life (Table 6). All other microbiota results in the relative abundance analysis on family and genus levels are listed in Supplemental Tables S5–8. The taxonomic distribution at genus level at the ages of 3 and 6 weeks is shown in Supplemental Fig. S3.

**Table 5:** Microbiota differences in infants at family and genus level.

Family			Genus		
	p_FDR	Fold Change		p_FDR	Fold Change
**Taxon (Infection episode in the first 3 months)**					
Bacteroidetes_Bacteroidia_Bacteroidales_*Prevotellaceae*	0.0002	87.0	Bacteroidetes_Bacteroidia_Bacteroidales_*Rikenellaceae_Alistipes*	5.67e-07	61.7
Bacteroidetes_Bacteroidia_Bacteroidales_*Rikenellaceae*	3.29e-07	61.7	Verrucomicrobia_Verrucomicrobiae_Verrucomicrobiales_*Verrucomicrobiaceae_Akkermansia*	5.67e-07	28.6
Verrucomicrobia_Verrucomicrobiae_Verrucomicrobiales_*Verrucomicrobiaceae*	3.29e-07	28.6	Firmicutes_Clostridia_Clostridiales_*FamilyXIIncertaeSedis_Peptoniphilus*	0.001	13.8
Actinobacteria_Actinobacteria_Actinomycetales_*Actinomycetaceae*	0.017	3.95	Firmicutes_Negativicutes_Selenomonadales_*Veillonellaceae_uncultured*	0.1	9.01
Actinobacteria_Coriobacteriia_Coriobacteriales_*Coriobacteriaceae*	0.039	3.16	Proteobacteria_Gammaproteobacteria_Enterobacteriales_*Enterobacteriaceae_Serratia*	0.0001	8.41
Actinobacteria_Actinobacteria_Micrococcales_*Micrococcaceae*	0.012	3.12	Bacteroidetes_Bacteroidia_Bacteroidales_*Porphyromonadaceae_Macellibacteroides*	0.056	7.06
			Proteobacteria_Gammaproteobacteria_Enterobacteriales_*Enterobacteriaceae_Klebsiella*	0.012	4.39
			Firmicutes_Clostridia_Clostridiales_*Ruminococcaceae_Faecalibacterium*	0.004	4.03
			Actinobacteria_Actinobacteria_Actinomycetales_*Actinomycetaceae_Actinomyces*	0.018	3.95
			Actinobacteria_Coriobacteriia_Coriobacteriales_*Coriobacteriaceae_Collinsella*	0.1	3.32
			Actinobacteria_Actinobacteria_Micrococcales_*Micrococcaceae_Rothia*	0.012	3.12
**Taxon (Infection episode in the first 6 months)**					
Bacteroidetes_Bacteroidia_Bacteroidales_*Prevotellaceae*	2.22e-06	44.8	Firmicutes_Clostridia_Clostridiales_*FamilyXIIncertaeSedis_Peptoniphilus*	0.0005	6.63
			Firmicutes_Clostridia_Clostridiales_*Ruminococcaceae_Faecalibacterium*	4.54e-09	4.71
			Proteobacteria_Gammaproteobacteria_Enterobacteriales_*Enterobacteriaceae_Proteus*	0.034	2.4
			Firmicutes_Clostridia_Clostridiales_*Lachnospiraceae_Anaerostipes*	3.14e-06	0.098

FDR-corrected *P* values for microbiota differences at 3 weeks of age between infants who developed a respiratory tract infection episode in the first 3 and 6 months of life and infants remaining healthy.

Standard Benjamini-Hochberg corrections for false discovery rate were applied.

*FDR* false discovery rate.

**Table 6:** Microbiota differences in infants at family and genus level.

Family			Genus		
	p_FDR	Fold Change		p_FDR	Fold Change
**Taxon (Infection episode in the first 3 months)**					
Bacteroidetes_Bacteroidia_Bacteroidales_*Prevotellaceae*	7.79e-06	168	Bacteroidetes_Bacteroidia_Bacteroidales_*Prevotellaceae_Prevotella*	0.0004	68.6
Firmicutes_Erysipelotrichia_Erysipelotrichales_*Erysipelotrichaceae*	0.001	4.87	Bacteroidetes_Bacteroidia_Bacteroidales_*Porphyromonadaceae_Macellibacteroides*	0.0004	23.0
Firmicutes_Clostridia_Clostridiales_*Ruminococcaceae*	0.004	3.42	Actinobacteria_Coriobacteriia_Coriobacteriales_*Coriobacteriaceae_Atopobium*	0.097	9.52
			Firmicutes_Clostridia_Clostridiales_*Ruminococcaceae_Subdoligranulum*	0.002	6.37
			Firmicutes_Erysipelotrichia_Erysipelotrichales_*Erysipelotrichaceae_Catenibacterium*	0.0004	4.79
			Firmicutes_Clostridia_Clostridiales_*Ruminococcaceae_Faecalibacterium*	0.028	2.76
			Firmicutes_Clostridia_Clostridiales_*Lachnospiraceae_Anaerostipes*	0.0002	0.034
**Taxon (Infection episode in the first 6 months)**					
			Firmicutes_Clostridia_Clostridiales_*Lachnospiraceae_Blautia*	0.093	0.342
			Firmicutes_Clostridia_Clostridiales_*Lachnospiraceae_Anaerostipes*	7.66e-07	0.046

FDR-corrected *P* values for microbiota differences at 6 weeks of age between infants who developed a respiratory tract infection episode in the first 3 and 6 months of lifeand infants remaining healthy.

Standard Benjamini-Hochberg corrections for false discovery rate were applied.

*FDR* false discovery rate.

### Early gut microbiota composition and RTI episodes in cases and controls matched for environmental and perinatal factors (sensitivity analysis)

**Fig. 5:**
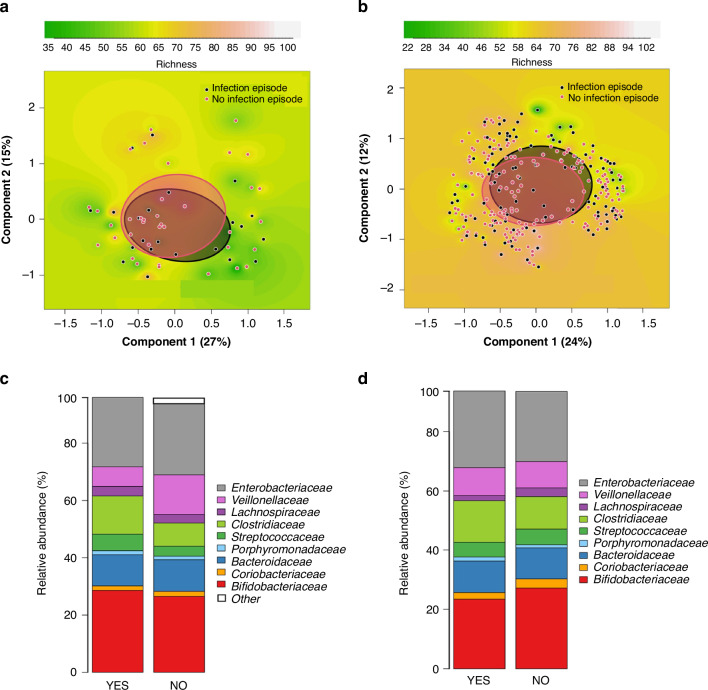
Principal coordinate analyses (PCoA) plots are based on Bray–Curtis dissimilarities of the samples, and show richness as the background at the time point of three weeks of age. The comparisons are between infants who developed an RTI episode during the first 3 (**a**) and 6 months of life (**b**). Clustered stacked column graphs demonstrate microbiota differences at the family level. The comparisons are between infants who developed an RTI episode (YES) in the first 3 (**c**) and 6 (**d**) months of life and carefully matched infants remaining healthy (NO) for the first 6 months of life. Infants with any infections before the 3-week stool sample were excluded.

After excluding infants with any infection symptoms before obtaining the early fecal samples, we compared the gut microbiota composition in infants later developing RTIs to that in controls matched for environmental and perinatal covariates. We observed no statistically significant differences in the overall composition of microbiota at 3 weeks of age based on beta diversity between the groups (Fig. 5).

The gut microbiota composition was highly similar at 3 weeks of age between infants who developed an RTI episode within the first 3 (*p* = 0.55) or 6 months (*p* = 0.30) of life and matched controls as visualized by PCoA at family level (Fig. 5a, b) and at genus level within the first 3 (*p* = 0.66) or 6 months of life (*p* = 0.25) (Supplemental Fig. S4a, b). The bacterial richness and diversity defined by the number of OTUs did not differ at 3 weeks of age between infants developing an RTI episode within the first 3 or 6 months of life and matched controls (Supplemental Table S2).

The taxonomic distribution of the dominant taxa was comparable to the unmatched analysis and the two most predominant bacteria of the microbiota at family level were *Bifidobacteriaceae* and *Enterobacteriaceae* at the age of 3 weeks (Fig. 5c and d). In the relative abundance analysis, the significant findings at family level were that in infants developing an RTI during the first 6 months of life had a higher abundance of *Acidaminococcaceae* and a lower abundance of *Veillonellaceae* compared to infants in the control group (Table 7). No such observation was made between infants developing infections in the first 3 months of life and matched controls. At genus level in the infant group developing RTIs within the first 3 months of life altogether 8 genera (*Dialister*, *Faecalibacterium*, *Roseburia*, *Proteus*, *Ruminococcaceae uncultured*, *Serratia*, *Pseudobutyrivibrio*, and *Rothia*) were increased, and 2 genera (*Akkermansia* and *Salmonella*) were less abundant (Table 7). Whereas in the infant group developing RTIs in the first 6 months of life altogether 6 genera (*Faecalibacterium*, *Proteus*, *Peptoniphilus*, *Pseudobutyrivibrio*, *Roseburia*, and *Anaerospora*) were increased, and 2 genera (*Anaerostipes* and *Veillonella*) were less abundant (Table 7). The most significant findings at genus level were that a higher abundance of butyrate-producing genera *Roseburia, Pseudobutyrivibrio*, and *Faecalibacterium*, and also genus *Proteus* was found both in infants who developed an RTI episode within the first 3 and 6 months of life (Table 7). All other results of the relative abundances of the sensitivity analysis on family and genus levels are listed in Supplemental Tables S9–10. The taxonomic distribution at genus level is shown in Supplemental Fig. S5.

**Table 7:** Microbiota differences in infants at family and genus level.

Family			Genus		
	p_FDR	Fold Change		p_FDR	Fold Change
**Taxon (Infection episode in the first 3 months)**					
No differences			Firmicutes_Negativicutes_Selenomonadales_*Veillonellaceae_Dialister*	1.02e-09	597
			Proteobacteria_Gammaproteobacteria_Enterobacteriales_*Enterobacteriaceae_Serratia*	0.059	179
			Firmicutes_Clostridia_Clostridiales_*Ruminococcaceae_uncultured*	0.007	64.7
			Firmicutes_Clostridia_Clostridiales_*Lachnospiraceae_Roseburia*	0.0002	58.4
			Proteobacteria_Gammaproteobacteria_Enterobacteriales_*Enterobacteriaceae_Proteus*	0.001	35.0
			Firmicutes_Clostridia_Clostridiales_*Ruminococcaceae_Faecalibacterium*	8.08e-05	25.4
			Firmicutes_Clostridia_Clostridiales_*Lachnospiraceae_Pseudobutyrivibrio*	0.039	8.73
			Actinobacteria_Actinobacteria_Micrococcales_*Micrococcaceae_Rothia*	0.062	4.14
			Verrucomicrobia_Verrucomicrobiae_Verrucomicrobiales_*Verrucomicrobiaceae_Akkermansia*	0.043	0.213
			Proteobacteria_Gammaproteobacteria_Enterobacteriales_*Enterobacteriaceae_Salmonella*	0.018	0.176
**Taxon (Infection episode in the first 6 months)**					
Firmicutes_Negativicutes_Selenomonadales_*Acidaminococcaceae*	0.002	3.18	Proteobacteria_Gammaproteobacteria_Enterobacteriales_*Enterobacteriaceae_Proteus*	4.99e-06	12.8
Firmicutes_Negativicutes_Selenomonadales_V*eillonellaceae*	0.091	0.90	Firmicutes_Clostridia_Clostridiales_*Ruminococcaceae_Faecalibacterium*	7.33e-16	11.1
			Firmicutes_Clostridia_Clostridiales_F*amilyXIIncertaeSedis_Peptoniphilus*	0.0004	8.61
			Firmicutes_Negativicutes_Selenomonadales_*Veillonellaceae_Anaerospora*	0.042	7.51
			Firmicutes_Clostridia_Clostridiales_*Lachnospiraceae_Roseburia*	0.004	4.39
			Firmicutes_Clostridia_Clostridiales_*Lachnospiraceae_Pseudobutyrivibrio*	0.0004	3.06
			Firmicutes_Negativicutes_Selenomonadales_*Veillonellaceae_Veillonella*	0.079	0.914
			Firmicutes_Clostridia_Clostridiales_*Lachnospiraceae_Anaerostipes*	1.58e-06	0.081

FDR-corrected *P* values for microbiota differences at 3 weeks of age between infants who developed a respiratory tract infection episode in the first 3 and 6 months of life with the exclusion of infants with any infection before the first fecal sample and matched infants remaining healthy.

Standard Benjamini-Hochberg corrections for false discovery rate were applied.

*FDR* false discovery rate.

When comparing the results to the unmatched analysis, we found similarities in the relative abundances at genus level that persisted when the groups were matched. Infants developing RTIs in the first 6 months of life had a lower abundance of a butyrate producer genus *Anaerostipes* in the samples taken at 3 and 6 weeks of age (Tables 5–7). We also observed that in the samples taken at 3 and 6 weeks of age, the relative abundance of another major butyrate producer genus *Faecalibacterium* was higher in infants developing RTIs during the first 3 and 6 months of life in both unmatched and matched analysis (Tables 5–7). The analysis also revealed that in the samples taken at 3 weeks of age, the genera *Serratia* and *Rothia* were more abundant in infants developing RTIs within the first 3 months of life and *Peptoniphilus* and *Proteus* were more abundant in infants developing RTIs within the first 6 months of life (Tables 5–7).

## Discussion

This prospective study demonstrated that alterations in the relative abundance of certain taxa of the maternal and early-life gut microbiota are associated with the occurrence of RTIs within the first 3 and 6 months of life. However, no association between maternal or early-life overall gut microbiota composition, measured with beta diversity, and the occurrence of RTIs in infants was found. We did observe an association in higher abundances of *Prevotellaceae* and adult-type butyrate producers, including *Faecalibacterium* spp., and some *Enterobacteriaceae* at three and to some extent also at 6 weeks of age in infants with RTIs compared to controls.

The gut microbiota of mothers of infants with infections was enriched in relative abundance of *Enterobacter* and *Citrobacter*, members of phylum Proteobacteria, and *Enterococcus*, which are all considered opportunistic pathogens. The *Enterobacteriaceae* family, a part of the Proteobacteria phylum found in the healthy human gut but with low abundance,^33^ comprises both commensal and opportunistic disease-causing pathogens. It has been suggested that the enrichment of *Enterobacteriaceae* in the gut is a marker for an unstable microbial composition, which is reportedly associated with various diseases such as inflammatory bowel disease, colorectal cancer, or metabolic syndrome.^34^ It has been proposed that perturbations to the maternal or neonatal microbiota might increase and prolong the window of risk for bronchiolitis in infancy.^35^ In previous studies, there has been limited data on the associations between maternal microbiota and infections in infants. In one prospective study, the composition of the maternal gut microbiota with higher counts of enterococci was shown to be associated with the increased risk of infant wheezing in the first 6 months of life.^36^ In line with this, we found here that the mothers of infants with RTIs showed an increased relative abundance of *Enterococcus* spp. in their gut microbiota around delivery.

The evidence from prior case-control studies evaluating bacterial profiles in infants and RTIs show inconsistent results.^20,21,23^ Hasegawa et al. found in 40 infants hospitalized with bronchiolitis that at 3 months of age, the likelihood of bronchiolitis was associated with a microbiota profile dominated by *Bacteroides*,^21^ whereas Li et al., based on analysis of 26 children, mostly aged from 2 to 6 years, found an association between recurrent RTIs and a lower alfa-diversity, and also a lower relative abundance of genera *Faecalibacterium, Bifidobacterium*, and *Eubacterium*, and a higher abundance of *Enterococcus*.^23^ Our results do not support these observed changes in the composition of the bacterial profile. Instead, we found genus *Faecalibacterium* to be more abundant in infants developing RTIs compared to healthy controls. A retrospective case-control study design cannot exclude the possibility that the infections per se result in a deviation in the gut microbiota. In our prospective cohort, we were able to avoid bias due to reverse causation by excluding all infants with any infections before the first gut microbiota samples were obtained at 3 weeks of age. In addition, we were able to match the controls for clinically important covariates such as mode of delivery and perinatal antibiotics, which are known to influence the early gut microbial composition.^37,38^ We did observe that infants developing RTIs in the first 6 months of life had a lower abundance of *Anaerostipes* spp. in the samples taken at 3 and 6 weeks of age (Tables 5–7). *Anaerostipes* spp. are known to produce butyrate from sugars but notably also from lactate and acetate, that are abundant short-chain fatty acids in early life produced by *Bifidobacteria* from breast milk.^39,40^ Hence the depletion of *Anaerostipes* spp. in the the gut of RTI infants may point towards an increased level of lactate and reduced levels of butyrate while there are also indications that this is related to a deviating immune response.^41^

The results from birth cohorts focusing on the relation between gut microbiota and RTIs have been variable. A Danish birth cohort assessed wheezing at 3 years of age and found no difference in alfa- or beta-diversity or the relative abundance of the gut microbiota at 9 months of age.^24^ In a prospective birth cohort from the USA, Moroishi et al. observed that higher gut microbiome diversity at 6 weeks of age increased the odds for RTIs by the age of 1 year.^25^ In a well-executed prospective birth cohort from the Netherlands, Reyman et al. found that enrichment of *Bifidobacterium* spp. and reduction of *Enterococcus* and *Klebsiella* spp. at 1 week of life was associated with the number of RTIs in the first year of life.^22^ Similarly, a recent study in the UK described a correlation with the microbiota in the first week of life and hospital admissions for viral LRTIs, suggesting a protective role for *Bifidobacterium* spp.^42^ We did not observe this in our HELMi cohort, where we monitored mild RTIs treated at home and where virtually all infants were breast-fed and contained *Bifidobacterium* spp. In contrast, we observed here that the gut microbiota of infants with infections contained an increased relative abundance of fecal butyrate-producing bacteria, including *Faecalibacterium*, *Ruminococcus*, and *Roseburia* spp. In healthy infants, these butyrate producers are linked to the conversion of solid foods as these are found to develop from around 4 to 6 months of age when weaning off breastmilk.^43^ We consider their increased relative abundance in infants with infections at 3 weeks and, to some extent, also at 6 weeks of age as reflecting a premature gut microbiota maturation.

The strength of our study lies in the large prospective longitudinal birth cohort of full-term infants born healthy. The follow-up starting at birth provided us with a large scale of weekly information on the occurrence of common RTIs mainly treated at home. The information on symptoms provided by parents gave a more comprehensive understanding of the real rate of common RTIs, whilst making our study hard to be comparable to other studies with a more severe outcome or an outcome defined by a healthcare professional. The fecal samples available enabled us the opportunity to study not only the microbiota of infants but also the maternal gut microbiota.

The main limitation of this work is that the cohort represented highly educated families in a high-income country. Although the development of infant microbiota appears universal, the pattern is affected by local culture and feeding practices.^44^ Almost all the mothers breastfed their infants, for instance, and very few reported smoking. Thus, the results may not be generalizable to child cohorts with different background characteristics. Moreover, our HELMi cohort included only infants who were born healthy, term, and singleton with birth weight exceeding 2.5 kg. The current methodology did not allow assessment of the functional properties of gut microbiota warranting further studies on the metabolic pathways of the microbiota and their possible associations with susceptibility to RTIs in early life.

## Conclusion

This study reported the association between maternal and infant gut microbiota and early-life RTIs in the offspring using a prospective online follow-up for respiratory symptoms.

This prospective study demonstrated that alterations in the relative abundance of taxa of the maternal and early-life gut microbiota are associated with infants’ early RTIs in full-term infants. Ideally, data on gut microbiota from observational cohort studies could be used in planning intervention studies in the future.

## Supplementary information

Supplemental Tables

## Data Availability

Sequencing data are accessible in ENA along with limited metadata (Study ID: PRJEB55243). Additional data can be obtained from the corresponding author upon reasonable request.
