# Supplementary material for: The association of maternal and infant early gut microbiota with respiratory infections in infants
Source: Pediatr Res. 2025 Aug 20;99(3):970–83. doi: 10.1038/s41390-025-04326-0 (PMC13021512; doi:10.1038/s41390-025-04326-0)
Supplement: Supplementary file 1 — Supplemental Tables [file 41390_2025_4326_MOESM1_ESM.pdf]

| **Supplemental Table S1** Baseline Characteristics of Study Population in the sensitivity analysis. | | | | |
|---|---|---|---|---|
| | categorical: n (%)<br>numeric: mean (SD) | | | |
| | Infants with RTI in the first 3 months (n=22) | Infants with no RTI in the first 3 months (n=37) | Infants with RTI in the first 6 months (n=109) | Infants with no RTI in the first 6 months (n=182) |
| Sex, male | 13 (59.1%) | 24 (64.9%) | 53 (48.60%) | 90 (49.5 %) |
| Year of birth | | | | |
| 2016 | 9 (40.9%) | 14 (37.8%) | 40 (36.7%) | 63 (34.6%) |
| 2017 | 11 (50.0%) | 21 (56.8%) | 63 (57.8%) | 111 (61.0%) |
| 2018 | 2 (9.1%) | 2 (5.4%) | 6 (5.5%) | 8 (4.4%) |
| Season of birth | | | | |
| Winter | 5 (22.7%) | 7 (18.9%) | 15 (13.8%) | 25 (13.7%) |
| Spring | 4 (18.2%) | 7 (18.9%) | 26 (23.9%) | 48 (26.4%) |
| Summer | 7 (31.8%) | 12 (32.4%) | 42 (38.5%) | 62 (34.1%) |
| Autumn | 6 (27.3%) | 11 (29.7%) | 26 (23.9%) | 47 (25.8%) |
| Mode of delivery | | | | |
| Caesarean delivery | 4 (18.2%) | 6 (16.2%) | 20 (18.3%) | 32 (17.6%) |
| - Mother received antibiotics | 4 (100%) | 6 (100%) | 20 (100%) | 32 (100%) |
| Vaginal birth | 18 (81.8%) | 31 (83.8%) | 89 (81.7%) | 150 (82.4%) |
| - Mother received antibiotics | 6 (33.3%) | 8 (25.8%) | 23 (25.8%) | 37 (20.3%) |
| Number of siblings in household | | | | |
| 0 siblings | 7 (31.8%) | 12 (32.4%) | 50 (45.9%) | 85 (46.7%) |
| ≥1 sibling | 15 (68.2%) | 25 (67.6%) | 59 (54.1%) | 97 (53.3%) |
| Breastfeeding | 22 (100%) | 36 (97.3%) | 107 (98.2%) | 181 (99.5%) |
| Exclusive (months) | 3.3 (1.8SD) | 3.7 (1.7SD) | 3.3 (2.0 SD) | 3.5 (1.7 SD) |
| Partial (months) | 11.0 (1.8SD) | 10.6 (3.1SD) | 10.6 (2.9SD) | 10.5 (2.9SD) |
| Any use of probiotics during pregnancy | 15 (68.2%) | 28 (75.7%) | 83 (76.1%) | 129 (70.9%) |
| Antibiotic use before 3-week fecal sample | 0 (0.0%) | 0 (0.0%) | 1 (0.9%) | 2 (1.1%) |
| Antibiotic use before 6-week fecal sample | 0 (0.0%) | 0 (0.0%) | 1 (0.9%) | 2 (1.1%) |
| Antibiotic use in the first 6 months of life | | | 3 (2.8%) | 3 (1.7%) |
| Vaccination at 12 months[a] | | | | |
| Full program | 14 (63.3%) | 27 (73%) | 88 (80.7%) | 142 (78.0%) |
| Partial program | 3 (13.6%) | 6 (16.2%) | 9 (8.3%) | 13 (7.1%) |
| No vaccination | 2 (9.1%) | 3 (8.1%) | 5 (4.6%) | 9 (4.9%) |
| Furry pet at home | 10 (45.5%) | 16 (43.2%) | 32 (29.4%) | 61 (33.5%) |
| Mother's age | 32.3 (3.5SD) | 34.3 (4.5SD) | 32.9 (4.0 SD) | 33.2 (4.2 SD) |
| Father's age | 34.1 (4.1SD) | 37.0 (6.0SD) | 34.6 (4.8 SD) | 35.2 (5.5 SD) |
| Maternal education | | | | |

| | | | | |
|---|---|---|---|---|
| Secondary school/upper secondary/vocational school | 5(22.7%) | 7 (18.9%) | 12 (11.0%) | 22 (12.1%) |
| University including polytechnic | 17 (77.3%) | 30 (81.1%) | 97 (89.0%) | 160 (87.9%) |
| Paternal education | | | | |
| Secondary school/upper secondary/vocational school | 4 (18.2%) | 13 (35.1%) | 24 (22.0%) | 43 (23.6%) |
| University including polytechnic | 18 (81.8%) | 24 (64.9%) | 84 (77.1%) | 135 (74.2%) |
| Maternal smoking | 0 (0.0%) | 0 (0.0%) | 0 (0.0%) | 0 (0.0%) |
| Paternal smoking | 1 (4.5%) | 6 (16.2%) | 15 (13.8%) | 28 (15.4%) |
| At least one parent with asthma | 2 (9.1%) | 4 (10.8%) | 16 (14.7%) | 24 (13.2%) |
| Mother with asthma | 2 (9.1%) | 3 (8.1%) | 11 (10.1%) | 16 (8.8%) |
| Father with asthma | 0 (0.0%) | 1 (2.7%) | 5 (4.6%) | 9 (4.9%) |
| At least one parent with autoimmune disease | 4 (18.2%) | 6 (16.2%) | 12 (11.0%) | 30 (16.5%) |
| Mother with autoimmune disease | 2 (9.1%) | 3 (8.1%) | 9 (8.3%) | 15 (8.2%) |
| Thyroid disease | 1 (4.5%) | 1 (2.7%) | 6 (5.5%) | 4 (2.2%) |
| Celiac disease | 0 (0.0%) | 1 (2.7%) | 1 (0.9%) | 4 (2.2%) |
| Other[b] | 1 (4.5%) | 0 (0.0%) | 1 (0.9%) | 10 (5.5%) |
| Father with autoimmune disease | 2 (9.1%) | 4 (10.8%) | 4 (3.7%) | 17 (9.3%) |
| Inflammatory bowel disease | 1 (4.5%) | 1 (2.7%) | 2 (1.2%) | 4 (2.2%) |
| Type 1 diabetes | 0 (0.0%) | 2 (5.4%) | 1 (0.9%) | 5 (2.7%) |
| Other[c] | 1 (4.5%) | 1 (2.7%) | 2 (1.8%) | 8 (4.4%) |

RTI, respiratory tract infection with fever or otitis media or a lower respiratory tract infection
Data missing (n): Vaccination (7-18-3-1), Father's age (1-2-0-0), Paternal smoking (1-3-0-0) Paternal education (1-4-1-0).
[a]The national immunization program: rotavirus, pneumococcus, diphtheria-tetanus-pertussis-polio-Hib, measles-mumps-rubella, and varicella-zoster virus.
[b]Inflammatory bowel disease, Type 1 diabetes, Psoriasis, Rheumatic disease
[c]Thyroid disease, Type 1 diabetes, Psoriasis, Rheumatic disease

**Supplemental Table S2.** Median number of OTUs in different subgroups and pairwise comparison.

| | Median number of OTUs (IQR) | p-values | Adjusted p-values |
|---|---|---|---|
| Mothers of infants with RTI in the first six months of life | 45 (38-54) | | |
| Mothers of controls | 48 (39-60) | 0.078* | 0.396* |
| Samples at three weeks of age | | | |
| Infants with RTI in the first 3 months of life | 11 (9-15) | | |
| Infants with RTI in the 6 months of life | 13 (10-17) | | |
| Controls with no RTI in the first 6 months of life | 13 (10-16) | 0.811** | 0.811** |
| | | 0.114† | 0.396† |
| Samples at six weeks of age | | | |
| Infants with RTI in the first 3 months of life | 13 (11-17.5) | | |
| Infants with RTI in the 6 months of life | 14 (11-19) | | |
| Controls with no RTI in the first 6 months of life | 13 (10-18) | 0.519†† | 0.664†† |
| | | 0.226‡ | 0.396‡ |
| Samples in the sensitivity analysis at three weeks of age | | | |
| Infants with RTI in the first 3 months of life. | 12.5 (10-14.3) | | |
| Matched controls with no RTI in the first 3 months of life. | 12 (9.5-15) | 0.201‡‡ | 0.396‡‡ |
| Infants with RTI in the first 6 months of life. | 13 (10-17) | | |
| Matched controls with no RTI in the first 6 months of life. | 13 (10-19) | 0.569§ | 0.664§ |

Pairwise comparisons (t-test) were made between subgroups as indicated in the table. Standard Benjamini-Hochberg corrections for false discovery rate (FDR) were applied.

OTU, operational taxonomic unit.

RTI, respiratory tract infection with fever or otitis media or a lower respiratory tract infection.

*The comparison is between mothers of infants with RTI in the first 6 months of life and Mothers of controls.

**The comparison is between infants with RTI in the first 3 months of life and Controls with no RTI in the first 6 months of life

†The comparison is between infants with RTI in the first 6 months of life and Controls with no RTI in the first 6 months of life

††The comparison is between infants with RTI in the first 3 months of life and Controls with no RTI in the first 6 months of life

‡The comparison is between infants with RTI in the first 6 months of life and Controls with no RTI in the first 6 months of life

‡‡The comparison is between infants with RTI in the first 3 months of life and Controls with no RTI in the first 3 months of life

§The comparison is between infants with RTI in the first 3 months of life and Controls with no RTI in the first 6 months of life

| **Supplemental Table S3. Maternal microbiota differences at family level.** P values for microbiota differences between mothers of infants with a respiratory tract infection episode in the first six months of life and mothers of randomly selected infants remaining healthy in the first six months of life. | | | |
|---|---|---|---|
| **Taxon** | 1_p | 1_p_FDR | FoldChange_1 |
| Actinobacteria_Actinobacteria_Actinomycetales_*Actinomycetaceae* | 0.547750322354224 | 0.771187329919644 | 0.900454697749334 |
| Actinobacteria_Actinobacteria_Bifidobacteriales_*Bifidobacteriaceae* | 0.48708804015511 | 0.771187329919644 | 0.824366583395851 |
| Actinobacteria_Actinobacteria_Micrococcales_*Micrococcaceae* | 0.17936368907015 | 0.521785277294982 | 1.35119953935527 |
| Actinobacteria_Actinobacteria_Streptomycetales_*Streptomycetaceae* | 0.855458926484446 | 0.900026791329953 | 0.86108715906358 |
| Actinobacteria_Coriobacteriia_Coriobacteriales_*Coriobacteriaceae* | 0.495405358622508 | 0.771187329919644 | 1.08806883623279 |
| Bacteroidetes_Bacteroidia_Bacteroidales_*Bacteroidaceae* | 0.768453685476794 | 0.87823278340205 | 1.06206787970738 |
| Bacteroidetes_Bacteroidia_Bacteroidales_*Porphyromonadaceae* | 0.871900954100892 | 0.900026791329953 | 0.915875241540338 |
| Bacteroidetes_Bacteroidia_Bacteroidales_*Prevotellaceae* | 0.552793282757251 | 0.771187329919644 | 0.842110074945582 |
| Bacteroidetes_Bacteroidia_Bacteroidales_*Rikenellaceae* | 0.0648738261386686 | 0.345993739406232 | 1.48455312386413 |
| Firmicutes_Bacilli_Bacillales_*Bacillaceae* | 0.0764326375604744 | 0.349406343133597 | 1.82066431432933 |
| Firmicutes_Bacilli_Bacillales_*Staphylococcaceae* | 0.225768101949139 | 0.525550937980434 | 2.28162636024351 |
| Firmicutes_Bacilli_Lactobacillales_*Enterococcaceae* | 4.95336625861190e-13 | 1.58507720275581e-11 | 99.0424883694302 |
| Firmicutes_Bacilli_Lactobacillales_*Lactobacillaceae* | 0.22992853536644 | 0.525550937980434 | 1.39203098404282 |
| Firmicutes_Bacilli_Lactobacillales_*Streptococcaceae* | 0.622110705186213 | 0.796301702638353 | 1.08599712721551 |
| Firmicutes_Clostridia_Clostridiales_*Christensenellaceae* | 0.211426542619489 | 0.525550937980434 | 1.18354502571422 |
| Firmicutes_Clostridia_Clostridiales_*Clostridiaceae* | 0.00390183476261205 | 0.0416195708011952 | 0.545899106648916 |
| Firmicutes_Clostridia_Clostridiales_*FamilyXIIIIncertaeSedis* | 0.00866757464287331 | 0.0693405971429865 | 1.31037807345115 |
| Firmicutes_Clostridia_Clostridiales_*Lachnospiraceae* | 0.351493311869507 | 0.661634469401425 | 0.957208943918734 |
| Firmicutes_Clostridia_Clostridiales_*Peptostreptococcaceae* | 0.935457699134042 | 0.935457699134042 | 1.02000576396014 |
| Firmicutes_Clostridia_Clostridiales_*Ruminococcaceae* | 0.722807348603591 | 0.85666056130796 | 0.986886389673838 |
| Firmicutes_Erysipelotrichia_Erysipelotrichales_*Erysipelotrichaceae* | 0.650463020535333 | 0.800569871428102 | 0.875421712153126 |
| Firmicutes_Erysipelotrichia_Erysipelotrichales_*Erysipelotrichaceae.1* | 0.8525848213556 | 0.900026791329953 | 1.02343635680295 |
| Firmicutes_Negativicutes_Selenomonadales_*Acidaminococcaceae* | 0.557640991630672 | 0.771187329919644 | 0.923969755769374 |
| Firmicutes_Negativicutes_Selenomonadales_*Veillonellaceae* | 0.578390497439733 | 0.771187329919644 | 1.30042699373081 |
| Proteobacteria_Betaproteobacteria_Burkholderiales_*Alcaligenaceae* | 0.167077598053228 | 0.521785277294982 | 1.43348528626518 |
| Proteobacteria_Betaproteobacteria_Burkholderiales_*Burkholderiaceae* | 0.1212460242442 | 0.431096975090489 | 1.61330143653713 |
| Proteobacteria_Betaproteobacteria_Burkholderiales_*Oxalobacteraceae* | 0.379495335724669 | 0.674658374621634 | 1.37594316741893 |

| | | | |
|---|---|---|---|
| Proteobacteria_Deltaproteobacteria_Desulfovibrionales_*Desulfovibrionaceae* | 0.102040223174122 | 0.408160892696488 | 1.44789864632323 |
| Proteobacteria_Gammaproteobacteria_Enterobacteriales_*Enterobacteriaceae* | 6.75346500282212e-08 | 1.08055440045154e-06 | 3.56777575332086 |
| Proteobacteria_Gammaproteobacteria_Pasteurellales_*Pasteurellaceae* | 0.0142545784511836 | 0.0912293020875752 | 2.01433332325726 |
| Proteobacteria_Gammaproteobacteria_Pseudomonadales_*Pseudomonadaceae* | 0.315475283354154 | 0.630950566708307 | 1.43011454873466 |
| Verrucomicrobia_Verrucomicrobiae_Verrucomicrobiales_*Verrucomicrobiaceae* | 0.283933199232486 | 0.605724158362637 | 1.36883887221042 |

| **Supplemental Table S4. Maternal microbiota differences at genus level.** P values for microbiota differences between mothers of infants with a respiratory tract infection episode in the first six months of life and mothers of randomly selected infants remaining healthy in the first six months of life. | | | |
|---|---|---|---|
| **Taxon** | 1_p | 1_p_FDR | FoldChange_1 |
| Actinobacteria_Actinobacteria_Actinomycetales_*Actinomycetaceae_Actinomyces* | 0.547750322354224 | 0.827425676906013 | 0.900454697749334 |
| Actinobacteria_Actinobacteria_Bifidobacteriales_*Bifidobacteriaceae_Bifidobacterium* | 0.48708804015511 | 0.819733530992746 | 0.824366583395851 |
| Actinobacteria_Actinobacteria_Micrococcales_*Micrococcaceae_Rothia* | 0.17936368907015 | 0.589337835516207 | 1.35119953935527 |
| Actinobacteria_Actinobacteria_Streptomycetales_*Streptomycetaceae_Streptomyces* | 0.855458926484446 | 0.972203302310801 | 0.86108715906358 |
| Actinobacteria_Coriobacteriia_Coriobacteriales_*Coriobacteriaceae_Atopobium* | 0.391650754089866 | 0.73037573060002 | 1.11858596412272 |
| Actinobacteria_Coriobacteriia_Coriobacteriales_*Coriobacteriaceae_Collinsella* | 0.687612500953569 | 0.891293079709915 | 1.07757513838557 |
| Actinobacteria_Coriobacteriia_Coriobacteriales_*Coriobacteriaceae_Slackia* | 0.516311933156585 | 0.827425676906013 | 1.12381836807232 |
| Actinobacteria_Coriobacteriia_Coriobacteriales_*Coriobacteriaceae_uncultured* | 0.686793598257402 | 0.891293079709915 | 0.996829856869498 |
| Bacteroidetes_Bacteroidia_Bacteroidales_*Bacteroidaceae_Bacteroides* | 0.768453685476794 | 0.930233408735067 | 1.06206787970738 |
| Bacteroidetes_Bacteroidia_Bacteroidales_*Porphyromonadaceae_Barnesiella* | 0.880594391452888 | 0.972203302310801 | 0.87574979853169 |
| Bacteroidetes_Bacteroidia_Bacteroidales_*Porphyromonadaceae_Macellibacteroides* | 0.432667013230285 | 0.746350597822241 | 1.22456896700536 |
| Bacteroidetes_Bacteroidia_Bacteroidales_*Porphyromonadaceae_Odoribacter* | 0.246832181274409 | 0.630913598514226 | 1.2228029593246 |
| Bacteroidetes_Bacteroidia_Bacteroidales_*Porphyromonadaceae_Paludibacter* | 0.247692331706684 | 0.630913598514226 | 1.36636648703098 |
| Bacteroidetes_Bacteroidia_Bacteroidales_*Prevotellaceae_Prevotella* | 0.418775824293483 | 0.746350597822241 | 0.77827297591325 |
| Bacteroidetes_Bacteroidia_Bacteroidales_*Prevotellaceae_uncultured* | 0.113382117616784 | 0.488960382222383 | 2.38956717004245 |
| Bacteroidetes_Bacteroidia_Bacteroidales_*Rikenellaceae_Alistipes* | 0.0648738261386686 | 0.376703713690909 | 1.48455312386413 |
| Firmicutes_Bacilli_Bacillales_*Bacillaceae_Bacillus* | 0.0764326375604744 | 0.376703713690909 | 1.82066431432933 |
| Firmicutes_Bacilli_Bacillales_*Staphylococcaceae_Staphylococcus* | 0.225768101949139 | 0.630913598514226 | 2.28162636024351 |
| Firmicutes_Bacilli_Lactobacillales_*Enterococcaceae_Enterococcus* | 4.95336625861190e-13 | 3.41782271844221e-11 | 99.0424883694302 |
| Firmicutes_Bacilli_Lactobacillales_*Lactobacillaceae_Lactobacillus* | 0.22992853536644 | 0.630913598514226 | 1.39203098404282 |
| Firmicutes_Bacilli_Lactobacillales_*Streptococcaceae_Lactococcus* | 0.865369291377902 | 0.972203302310801 | 1.04054550469332 |
| Firmicutes_Bacilli_Lactobacillales_*Streptococcaceae_Streptococcus* | 0.613806313342876 | 0.864339502462418 | 1.08997747854971 |

| | | | |
|---|---|---|---|
| Firmicutes_Clostridia_Clostridiales_*Christensenellaceae_uncultured* | 0.211426542619489 | 0.630913598514226 | 1.18354502571422 |
| Firmicutes_Clostridia_Clostridiales_*Clostridiaceae_Clostridium* | 0.00390183476261205 | 0.0673066496550578 | 0.545899106648916 |
| Firmicutes_Clostridia_Clostridiales_*FamilyXIIIIncertaeSedis_IncertaeSedis* | 0.00866757464287331 | 0.119612530071652 | 1.31037807345115 |
| Firmicutes_Clostridia_Clostridiales_*Lachnospiraceae_Acetitomaculum* | 0.303367275225251 | 0.67523683840459 | 0.823299256861192 |
| Firmicutes_Clostridia_Clostridiales_*Lachnospiraceae_Anaerostipes* | 0.963247708431186 | 0.972203302310801 | 1.00500123967215 |
| Firmicutes_Clostridia_Clostridiales_*Lachnospiraceae_Blautia* | 0.354980736305488 | 0.73037573060002 | 0.92488815962784 |
| Firmicutes_Clostridia_Clostridiales_*Lachnospiraceae_Butyrivibrio* | 0.151149032233662 | 0.548909643374879 | 0.821822980968426 |
| Firmicutes_Clostridia_Clostridiales_*Lachnospiraceae_Coprococcus* | 0.0763139104869921 | 0.376703713690909 | 1.15982738433243 |
| Firmicutes_Clostridia_Clostridiales_*Lachnospiraceae_Defluviitalea* | 0.430044370509643 | 0.746350597822241 | 0.968409512405585 |
| Firmicutes_Clostridia_Clostridiales_*Lachnospiraceae_Dorea* | 0.967544489744447 | 0.972203302310801 | 0.99606655180792 |
| Firmicutes_Clostridia_Clostridiales_*Lachnospiraceae_Howardella* | 0.293683349721578 | 0.67523683840459 | 0.937552117652955 |
| Firmicutes_Clostridia_Clostridiales_*Lachnospiraceae_IncertaeSedis* | 0.948454215971677 | 0.972203302310801 | 1.0036327414315 |
| Firmicutes_Clostridia_Clostridiales_*Lachnospiraceae_Moryella* | 0.805384850651086 | 0.958130253360775 | 0.988598210722985 |
| Firmicutes_Clostridia_Clostridiales_*Lachnospiraceae_Pseudobutyrivibrio* | 0.256022909542005 | 0.630913598514226 | 0.81174560541043 |
| Firmicutes_Clostridia_Clostridiales_*Lachnospiraceae_Roseburia* | 0.574843236481873 | 0.843918793984027 | 0.95411905709736 |
| Firmicutes_Clostridia_Clostridiales_*Lachnospiraceae_uncultured* | 0.972203302310801 | 0.972203302310801 | 1.00265025070941 |
| Firmicutes_Clostridia_Clostridiales_*Peptostreptococcaceae_IncertaeSedis* | 0.935457699134042 | 0.972203302310801 | 1.02000576396014 |
| Firmicutes_Clostridia_Clostridiales_*Ruminococcaceae_Anaerofilum* | 0.023852853322382 | 0.182871875471596 | 1.43136538071218 |
| Firmicutes_Clostridia_Clostridiales_*Ruminococcaceae_Anaerotruncus* | 0.0210502452454775 | 0.181558365242244 | 1.68232386530885 |
| Firmicutes_Clostridia_Clostridiales_*Ruminococcaceae_Faecalibacterium* | 0.60937414559565 | 0.864339502462418 | 0.949627478738491 |
| Firmicutes_Clostridia_Clostridiales_*Ruminococcaceae_IncertaeSedis* | 0.723932993021392 | 0.891988866401359 | 1.03957981927889 |
| Firmicutes_Clostridia_Clostridiales_*Ruminococcaceae_Oscillibacter* | 0.915582695022615 | 0.972203302310801 | 1.03017232273235 |
| Firmicutes_Clostridia_Clostridiales_*Ruminococcaceae_Oscillospira* | 0.365839640433056 | 0.73037573060002 | 1.13578398686497 |
| Firmicutes_Clostridia_Clostridiales_*Ruminococcaceae_Ruminococcus* | 0.532760471022972 | 0.827425676906013 | 1.04131250006243 |
| Firmicutes_Clostridia_Clostridiales_*Ruminococcaceae_Sporobacter* | 0.0169721054782742 | 0.167296468285846 | 1.39510831562495 |

| | | | |
|---|---|---|---|
| Firmicutes_Clostridia_Clostridiales_*Ruminococcaceae_Subdoligranulum* | 0.188552285189941 | 0.591368530822996 | 0.88623139101582 |
| Firmicutes_Clostridia_Clostridiales_*Ruminococcaceae_uncultured* | 0.0657855268076911 | 0.376703713690909 | 1.16039305855306 |
| Firmicutes_Erysipelotrichia_Erysipelotrichales_*Erysipelotrichaceae_Catenibacterium* | 0.551617117937342 | 0.827425676906013 | 1.01015858397913 |
| Firmicutes_Erysipelotrichia_Erysipelotrichales_*Erysipelotrichaceae_Turicibacter* | 0.650463020535333 | 0.891293079709915 | 0.875421712153126 |
| Firmicutes_Erysipelotrichia_Erysipelotrichales_*Erysipelotrichaceae_uncultured* | 0.710451005565875 | 0.891293079709915 | 1.05173552851204 |
| Firmicutes_Negativicutes_Selenomonadales_*Acidaminococcaceae_Acidaminococcus* | 0.954943535649706 | 0.972203302310801 | 1.01419373032848 |
| Firmicutes_Negativicutes_Selenomonadales_*Acidaminococcaceae_Succiniclasticum* | 0.909468064493366 | 0.972203302310801 | 0.965155963802457 |
| Firmicutes_Negativicutes_Selenomonadales_*Acidaminococcaceae_uncultured* | 0.385858250173928 | 0.73037573060002 | 0.926097609465087 |
| Firmicutes_Negativicutes_Selenomonadales_*Veillonellaceae_Dialister* | 0.701448885897338 | 0.891293079709915 | 1.09119436122948 |
| Firmicutes_Negativicutes_Selenomonadales_*Veillonellaceae_Megamonas* | 0.127999396169301 | 0.490664351982319 | 2.63371957856635 |
| Firmicutes_Negativicutes_Selenomonadales_*Veillonellaceae_Selenomonas* | 0.0492530544066905 | 0.339846075406164 | 2.16352781945149 |
| Firmicutes_Negativicutes_Selenomonadales_*Veillonellaceae_uncultured* | 0.6693495229846 | 0.891293079709915 | 0.758609828896151 |
| Firmicutes_Negativicutes_Selenomonadales_*Veillonellaceae_Veillonella* | 0.537028756409207 | 0.827425676906013 | 0.952943484821603 |
| Proteobacteria_Betaproteobacteria_Burkholderiales_*Alcaligenaceae_Bordetella* | 0.167077598053228 | 0.576417713283635 | 1.43348528626518 |
| Proteobacteria_Betaproteobacteria_Burkholderiales_*Burkholderiaceae_Ralstonia* | 0.1212460242442 | 0.490664351982319 | 1.61330143653713 |
| Proteobacteria_Betaproteobacteria_Burkholderiales_*Oxalobacteraceae_Oxalobacter* | 0.379495335724669 | 0.73037573060002 | 1.37594316741893 |
| Proteobacteria_Deltaproteobacteria_Desulfovibrionales_*Desulfovibrionaceae_Desulfovibrio* | 0.102040223174122 | 0.469385026600961 | 1.44789864632323 |
| Proteobacteria_Gammaproteobacteria_Enterobacteriales_*Enterobacteriaceae_Citrobacter* | 3.80189978637959e-06 | 8.74436950867305e-05 | 3.2886686680753 |
| Proteobacteria_Gammaproteobacteria_Enterobacteriales_*Enterobacteriaceae_Enterobacter* | 7.05242684032395e-08 | 2.43308725991176e-06 | 3.50475700172365 |
| Proteobacteria_Gammaproteobacteria_Pasteurellales_*Pasteurellaceae_Haemophilus* | 0.0142545784511836 | 0.163927652188612 | 2.01433332325726 |
| Proteobacteria_Gammaproteobacteria_Pseudomonadales_*Pseudomonadaceae_Pseudomonas* | 0.315475283354154 | 0.680243579732394 | 1.43011454873466 |
| Verrucomicrobia_Verrucomicrobiae_Verrucomicrobiales_*Verrucomicrobiaceae_Akkermansia* | 0.283933199232486 | 0.67523683840459 | 1.36883887221042 |

| **Supplemental Table S5. Microbiota differences in infants at family level.** P values for microbiota differences at three weeks of age between infants who developed a respiratory tract infection episode in the first three and six months of life and randomly selected infants remaining healthy. | | | |
|---|---|---|---|
| **Taxon (Infection episode in the first 3 months)** | 1_p | 1_p_FDR | FoldChange_1 |
| Actinobacteria_Actinobacteria_Actinomycetales_*Actinomycetaceae* | 0.00286778452580426 | 0.0166331502496647 | 3.94488393434 |
| Actinobacteria_Actinobacteria_Bifidobacteriales_*Bifidobacteriaceae* | 0.62401963634519 | 0.829146552878862 | 0.939521095714248 |
| Actinobacteria_Actinobacteria_Micrococcales_*Micrococcaceae* | 0.00160280764184033 | 0.0116203554033424 | 3.11701980088676 |
| Actinobacteria_Coriobacteriia_Coriobacteriales_*Coriobacteriaceae* | 0.00810646125276185 | 0.0391812293883489 | 3.16290793223038 |
| Bacteroidetes_Bacteroidia_Bacteroidales_*Bacteroidaceae* | 0.799693274350743 | 0.927644198246862 | 0.922977473034378 |
| Bacteroidetes_Bacteroidia_Bacteroidales_*Porphyromonadaceae* | 0.583689121098809 | 0.829146552878862 | 1.34333709464104 |
| Bacteroidetes_Bacteroidia_Bacteroidales_*Prevotellaceae* | 2.07925014703717e-05 | 0.000200994180880259 | 86.9738188508774 |
| Bacteroidetes_Bacteroidia_Bacteroidales_*Rikenellaceae* | 1.92257903807174e-08 | 3.28523471656551e-07 | 61.6758300404305 |
| Firmicutes_Bacilli_Bacillales_*Bacillaceae* | 0.945892537070054 | 0.945892537070054 | 0.970894853005237 |
| Firmicutes_Bacilli_Bacillales_*FamilyXIIncertaeSedis* | 0.629007729770171 | 0.829146552878862 | 1.20601265185497 |
| Firmicutes_Bacilli_Bacillales_*Staphylococcaceae* | 0.487335655271 | 0.829146552878862 | 1.1933634195032 |
| Firmicutes_Bacilli_Lactobacillales_*Carnobacteriaceae* | 0.265783061574202 | 0.612350780982685 | 1.84474083806299 |
| Firmicutes_Bacilli_Lactobacillales_*Enterococcaceae* | 0.544331758485173 | 0.829146552878862 | 1.34135844825761 |
| Firmicutes_Bacilli_Lactobacillales_*Lactobacillaceae* | 0.932395004714291 | 0.945892537070054 | 1.03918711242885 |
| Firmicutes_Bacilli_Lactobacillales_*Streptococcaceae* | 0.701827208902294 | 0.848041210756939 | 1.09530095066718 |
| Firmicutes_Clostridia_Clostridiales_*Clostridiaceae* | 0.527777094747102 | 0.829146552878862 | 0.771376450430076 |
| Firmicutes_Clostridia_Clostridiales_*FamilyXIIncertaeSedis* | 0.157621604874637 | 0.492834888809513 | 2.12440553745944 |
| Firmicutes_Clostridia_Clostridiales_*Lachnospiraceae* | 0.857452428824903 | 0.945892537070054 | 0.931839053758197 |
| Firmicutes_Clostridia_Clostridiales_*Peptostreptococcaceae* | 0.105902810428533 | 0.383897687803432 | 4.47134427941515 |
| Firmicutes_Clostridia_Clostridiales_*Ruminococcaceae* | 0.169943065106729 | 0.492834888809513 | 1.66819317545847 |
| Firmicutes_Erysipelotrichia_Erysipelotrichales_*Erysipelotrichaceae* | 0.195537366649365 | 0.51550760298469 | 1.65670974436791 |
| Firmicutes_Negativicutes_Selenomonadales_*Acidaminococcaceae* | 0.274502074233617 | 0.612350780982685 | 2.31644557217682 |
| Firmicutes_Negativicutes_Selenomonadales_*Veillonellaceae* | 0.921894524933283 | 0.945892537070054 | 1.41791816490437 |
| Proteobacteria_Betaproteobacteria_Burkholderiales_*Burkholderiaceae* | 0.0906166236601032 | 0.375411726591856 | 0.145150431283708 |
| Proteobacteria_Betaproteobacteria_Burkholderiales_*Oxalobacteraceae* | 0.669885721805076 | 0.844638518797705 | 0.618736297542833 |
| Proteobacteria_Deltaproteobacteria_Desulfovibrionales_*Desulfovibrionaceae* | 0.451393547545737 | 0.829146552878862 | 0.448150960787017 |
| Proteobacteria_Gammaproteobacteria_Enterobacteriales_*Enterobacteriaceae* | 0.608321323770679 | 0.829146552878862 | 0.929386261590883 |

| | | | |
|---|---|---|---|
| Proteobacteria_Gammaproteobacteria_Pasteurellales_*Pasteurellaceae* | 0.537828702790945 | 0.829146552878862 | 1.33777539106372 |
| Verrucomicrobia_Verrucomicrobiae_Verrucomicrobiales_*Verrucomicrobiaceae* | 2.26567911487277e-08 | 3.28523471656551e-07 | 28.5996127541926 |
| **Taxon (Infection episode in the first 6 months)** | | | |
| Actinobacteria_Actinobacteria_Actinomycetales_*Actinomycetaceae* | 0.774789229697811 | 0.907999037703511 | 0.908337113188653 |
| Actinobacteria_Actinobacteria_Bifidobacteriales_*Bifidobacteriaceae* | 0.265830559014953 | 0.724133046414423 | 1.11755010174148 |
| Actinobacteria_Actinobacteria_Micrococcales_*Micrococcaceae* | 0.923796596802656 | 0.923796596802656 | 1.02507954605204 |
| Actinobacteria_Coriobacteriia_Coriobacteriales_*Coriobacteriaceae* | 0.152465062286049 | 0.724133046414423 | 1.56278600211559 |
| Bacteroidetes_Bacteroidia_Bacteroidales_*Bacteroidaceae* | 0.331132722050617 | 0.724133046414423 | 0.995331457354929 |
| Bacteroidetes_Bacteroidia_Bacteroidales_Po*rphyromonadaceae* | 0.374551575731598 | 0.724133046414423 | 1.24978081145249 |
| Bacteroidetes_Bacteroidia_Bacteroidales_*Prevotellaceae* | 7.64312802188211e-08 | 2.21650712634581e-06 | 44.8331428984065 |
| Bacteroidetes_Bacteroidia_Bacteroidales_*Rikenellaceae* | 0.360733944717324 | 0.724133046414423 | 1.84643208487819 |
| Firmicutes_Bacilli_Bacillales_*Bacillaceae* | 0.882687544255599 | 0.923796596802656 | 0.954196854738033 |
| Firmicutes_Bacilli_Bacillales_*FamilyXIIncertaeSedis* | 0.542700070626903 | 0.835734035119266 | 1.16076905828699 |
| Firmicutes_Bacilli_Bacillales_*Staphylococcaceae* | 0.21798764843239 | 0.724133046414423 | 1.24487773773262 |
| Firmicutes_Bacilli_Lactobacillales_*Carnobacteriaceae* | 0.707889008195246 | 0.907999037703511 | 1.2439092989594 |
| Firmicutes_Bacilli_Lactobacillales_*Enterococcaceae* | 0.101032042517163 | 0.724133046414423 | 1.76511916480728 |
| Firmicutes_Bacilli_Lactobacillales_*Lactobacillaceae* | 0.500973844121604 | 0.835734035119266 | 0.804214938267482 |
| Firmicutes_Bacilli_Lactobacillales_*Streptococcaceae* | 0.651108190236675 | 0.907999037703511 | 1.07974333351745 |
| Firmicutes_Clostridia_Clostridiales_*Clostridiaceae* | 0.227826269520595 | 0.724133046414423 | 0.700917922377601 |
| Firmicutes_Clostridia_Clostridiales_*FamilyXIIncertaeSedis* | 0.131654227575734 | 0.724133046414423 | 1.74057393146784 |
| Firmicutes_Clostridia_Clostridiales_*Lachnospiraceae* | 0.328680498199373 | 0.724133046414423 | 1.31794582291674 |
| Firmicutes_Clostridia_Clostridiales_*Peptostreptococcaceae* | 0.47875271709744 | 0.835734035119266 | 0.626926018449662 |
| Firmicutes_Clostridia_Clostridiales_*Ruminococcaceae* | 0.913599519064551 | 0.923796596802656 | 0.971803049237685 |
| Firmicutes_Erysipelotrichia_Erysipelotrichales_*Erysipelotrichaceae* | 0.54754988507814 | 0.835734035119266 | 0.847436565292345 |
| Firmicutes_Negativicutes_Selenomonadales_*Acidaminococcaceae* | 0.118074212179036 | 0.724133046414423 | 2.37708872084714 |
| Firmicutes_Negativicutes_Selenomonadales_*Veillonellaceae* | 0.0745008384408629 | 0.724133046414423 | 0.934436622143514 |
| Proteobacteria_Betaproteobacteria_Burkholderiales_*Burkholderiaceae* | 0.886899040315956 | 0.923796596802656 | 0.34094591385051 |
| Proteobacteria_Betaproteobacteria_Burkholderiales_*Oxalobacteraceae* | 0.782757791123717 | 0.907999037703511 | 0.800140187209349 |
| Proteobacteria_Deltaproteobacteria_Desulfovibrionales_*Desulfovibrionaceae* | 0.774523182068567 | 0.907999037703511 | 0.821186926845137 |
| Proteobacteria_Gammaproteobacteria_Enterobacteriales_Enterobacteriaceae | 0.31248939589058 | 0.724133046414423 | 0.907367556899126 |

| | | | |
|---|---|---|---|
| Proteobacteria_Gammaproteobacteria_Pasteurellales_*Pasteurellaceae* | 0.261333298614847 | 0.724133046414423 | 1.44758340234262 |
| Verrucomicrobia_Verrucomicrobiae_Verrucomicrobiales_*Verrucomicrobiaceae* | 0.717326657274052 | 0.907999037703511 | 1.18153977701517 |

| **Supplement Table S6. Microbiota differences in infants at genus level.** P values for microbiota differences at three weeks of age between infants who developed a respiratory tract infection episode in the first three and six months of life and randomly selected infants remaining healthy. | | | |
|---|---|---|---|
| **Taxon (Infection episode in the first 3 months)** | 1_p | 1_p_FDR | FoldChange_1 |
| Actinobacteria_Actinobacteria_Actinomycetales_*Actinomycetaceae_Actinomyces* | 0.00286778452580426 | 0.0179236532862766 | 3.94488393434 |
| Actinobacteria_Actinobacteria_Bifidobacteriales_*Bifidobacteriaceae_Bifidobacterium* | 0.62401963634519 | 0.850010445635366 | 0.939521095714248 |
| Actinobacteria_Actinobacteria_Micrococcales_*Micrococcaceae_Rothia* | 0.00160280764184033 | 0.0118369591510677 | 3.11701980088676 |
| Actinobacteria_Coriobacteriia_Coriobacteriales_*Coriobacteriaceae_Collinsella* | 0.0207789372614797 | 0.0987278512982762 | 3.3162995337386 |
| Actinobacteria_Coriobacteriia_Coriobacteriales_*Coriobacteriaceae_Slackia* | 0.0646244059722281 | 0.269268358217617 | 2.63200138291543 |
| Actinobacteria_Coriobacteriia_Coriobacteriales_*Coriobacteriaceae_uncultured* | 0.193735917076062 | 0.483772585939912 | 1.9028551593568 |
| Bacteroidetes_Bacteroidia_Bacteroidales_*Bacteroidaceae_Bacteroides* | 0.799693274350743 | 0.887252842026868 | 0.922977473034378 |
| Bacteroidetes_Bacteroidia_Bacteroidales_*Porphyromonadaceae_Barnesiella* | 0.852996270808294 | 0.90744284128542 | 1.11294521499336 |
| Bacteroidetes_Bacteroidia_Bacteroidales_*Porphyromonadaceae_Macellibacteroides* | 0.0100546538206615 | 0.0558591878925641 | 7.05475340475472 |
| Bacteroidetes_Bacteroidia_Bacteroidales_*Porphyromonadaceae_Paludibacter* | 0.816272614664719 | 0.887252842026868 | 1.16098585740517 |
| Bacteroidetes_Bacteroidia_Bacteroidales_*Rikenellaceae_Alistipes* | 1.92257903807174e-08 | 5.66419778718192e-07 | 61.6758300404305 |
| Firmicutes_Bacilli_Bacillales_*Bacillaceae_Bacillus* | 0.945892537070054 | 0.965196466398014 | 0.970894853005237 |
| Firmicutes_Bacilli_Bacillales_*FamilyXIIncertaeSedis_Gemella* | 0.629007729770171 | 0.850010445635366 | 1.20601265185497 |
| Firmicutes_Bacilli_Bacillales_*Staphylococcaceae_Staphylococcus* | 0.487335655271 | 0.812226092118333 | 1.1933634195032 |
| Firmicutes_Bacilli_Lactobacillales_*Carnobacteriaceae_Granulicatella* | 0.265783061574202 | 0.577789264291744 | 1.84474083806299 |
| Firmicutes_Bacilli_Lactobacillales_*Enterococcaceae_Enterococcus* | 0.544331758485173 | 0.850010445635366 | 1.34135844825761 |
| Firmicutes_Bacilli_Lactobacillales_*Lactobacillaceae_Lactobacillus* | 0.932395004714291 | 0.965196466398014 | 1.03918711242885 |
| Firmicutes_Bacilli_Lactobacillales_*Streptococcaceae_Streptococcus* | 0.701827208902294 | 0.887252842026868 | 1.09530095066718 |
| Firmicutes_Clostridia_Clostridiales_*Clostridiaceae_Clostridium* | 0.527777094747102 | 0.850010445635366 | 0.771376450430076 |
| Firmicutes_Clostridia_Clostridiales_*FamilyXIIncertaeSedis_Finegoldia* | 0.379737544851268 | 0.762955190972668 | 1.72444391826133 |
| Firmicutes_Clostridia_Clostridiales_*FamilyXIIncertaeSedis_Parvimonas* | 0.381477595486334 | 0.762955190972668 | 1.94568273128881 |

| | | | |
|---|---|---|---|
| Firmicutes_Clostridia_Clostridiales_*FamilyXIIncertaeSedis_Peptoniphilus* | 9.65819069262554e-05 | 0.00120727383657819 | 13.802053727334 |
| Firmicutes_Clostridia_Clostridiales_*Lachnospiraceae_Anaerostipes* | 0.203184486094763 | 0.483772585939912 | 0.533629359977676 |
| Firmicutes_Clostridia_Clostridiales_*Lachnospiraceae_Blautia* | 0.473384493171608 | 0.812226092118333 | 0.78902794919671 |
| Firmicutes_Clostridia_Clostridiales_*Lachnospiraceae_Coprococcus* | 0.174909818675154 | 0.483772585939912 | 2.10720290384338 |
| Firmicutes_Clostridia_Clostridiales_*Lachnospiraceae_Dorea* | 0.620872001992274 | 0.850010445635366 | 0.605738358450308 |
| Firmicutes_Clostridia_Clostridiales_*Lachnospiraceae_IncertaeSedis* | 0.769033310668351 | 0.887252842026868 | 0.873822398232766 |
| Firmicutes_Clostridia_Clostridiales_*Lachnospiraceae_uncultured* | 0.988383158102718 | 0.988383158102718 | 0.994970724595428 |
| Firmicutes_Clostridia_Clostridiales_*Peptostreptococcaceae_IncertaeSedis* | 0.105902810428533 | 0.330946282589166 | 4.47134427941515 |
| Firmicutes_Clostridia_Clostridiales_*Ruminococcaceae_Faecalibacterium* | 0.000349693232048704 | 0.00349693232048704 | 4.02647383976636 |
| Firmicutes_Clostridia_Clostridiales_*Ruminococcaceae_IncertaeSedis* | 0.620400395491326 | 0.850010445635366 | 0.689048041832079 |
| Firmicutes_Clostridia_Clostridiales_*Ruminococcaceae_Subdoligranulum* | 0.763507340909029 | 0.887252842026868 | 1.18499979098838 |
| Firmicutes_Clostridia_Clostridiales_*Ruminococcaceae_uncultured* | 0.0723282766696171 | 0.278185679498527 | 3.52181985036001 |
| Firmicutes_Erysipelotrichia_Erysipelotrichales_*Erysipelotrichaceae_Catenibacterium* | 0.762557595739987 | 0.887252842026868 | 1.38931842468598 |
| Firmicutes_Erysipelotrichia_Erysipelotrichales_*Erysipelotrichaceae_Solobacterium* | 0.430600848931396 | 0.806059906331674 | 2.41197316083513 |
| Firmicutes_Negativicutes_Selenomonadales_*Acidaminococcaceae_Acidaminococcus* | 0.441483654111876 | 0.806059906331674 | 1.99673804260551 |
| Firmicutes_Negativicutes_Selenomonadales_*Acidaminococcaceae_uncultured* | 0.228876259494502 | 0.520173317032959 | 2.59488435576618 |
| Firmicutes_Negativicutes_Selenomonadales_*Veillonellaceae_uncultured* | 0.0217201272856208 | 0.0987278512982762 | 9.01142785754974 |
| Firmicutes_Negativicutes_Selenomonadales_*Veillonellaceae_Veillonella* | 0.806396491704234 | 0.887252842026868 | 1.44010427158038 |
| Proteobacteria_Betaproteobacteria_Burkholderiales_*Burkholderiaceae_Ralstonia* | 0.0906166236601032 | 0.302055412200344 | 0.145150431283708 |
| Proteobacteria_Betaproteobacteria_Burkholderiales_*Oxalobacteraceae_Oxalobacter* | 0.669885721805076 | 0.881428581322468 | 0.618736297542833 |

| | | | |
|---|---|---|---|
| Proteobacteria_Deltaproteobacteria_Desulfovibrionales_*Desulfovibrionaceae_Desulfovibrio* | 0.451393547545737 | 0.806059906331674 | 0.448150960787017 |
| Proteobacteria_Gammaproteobacteria_Enterobacteriales_*Enterobacteriaceae_Citrobacter* | 0.083555654481352 | 0.298413051719114 | 0.665510716027772 |
| Proteobacteria_Gammaproteobacteria_Enterobacteriales_*Enterobacteriaceae_Enterobacter* | 0.81294861543008 | 0.887252842026868 | 0.940081315245915 |
| Proteobacteria_Gammaproteobacteria_Enterobacteriales_*Enterobacteriaceae_Klebsiella* | 0.00165717428114947 | 0.0118369591510677 | 4.38547437475644 |
| Proteobacteria_Gammaproteobacteria_Enterobacteriales_*Enterobacteriaceae_Proteus* | 0.157514977789449 | 0.463279346439556 | 1.82097197551722 |
| Proteobacteria_Gammaproteobacteria_Enterobacteriales_*Enterobacteriaceae_Salmonella* | 0.191154565895128 | 0.483772585939912 | 1.70158244476873 |
| Proteobacteria_Gammaproteobacteria_Enterobacteriales_*Enterobacteriaceae_Serratia* | 6.23663171288149e-06 | 0.000103943861881358 | 8.40946904350798 |
| Proteobacteria_Gammaproteobacteria_Pasteurellales_*Pasteurellaceae_Haemophilus* | 0.564322490828966 | 0.850010445635366 | 1.31477580621482 |
| Verrucomicrobia_Verrucomicrobiae_Verrucomicrobiales_*Verrucomicrobiaceae_Akkermansia* | 2.26567911487277e-08 | 5.66419778718192e-07 | 28.5996127541926 |
| **Taxon (Infection episode in the first 6 months)** | | | |
| Actinobacteria_Actinobacteria_Actinomycetales_*Actinomycetaceae_Actinomyces* | 0.774789229697811 | 0.943351769816377 | 0.908337113188653 |
| Actinobacteria_Actinobacteria_Bifidobacteriales_*Bifidobacteriaceae_Bifidobacterium* | 0.265830559014953 | 0.753357491279219 | 1.11755010174148 |
| Actinobacteria_Actinobacteria_Micrococcales_*Micrococcaceae_Rothia* | 0.923796596802656 | 0.943351769816377 | 1.02507954605204 |
| Actinobacteria_Coriobacteriia_Coriobacteriales_*Coriobacteriaceae_Collinsella* | 0.265001282000466 | 0.753357491279219 | 1.50568446486294 |
| Actinobacteria_Coriobacteriia_Coriobacteriales_*Coriobacteriaceae_Slackia* | 0.296707030113573 | 0.753357491279219 | 1.4708127615635 |
| Actinobacteria_Coriobacteriia_Coriobacteriales_*Coriobacteriaceae_uncultured* | 0.303600056377046 | 0.753357491279219 | 1.4408942051076 |
| Bacteroidetes_Bacteroidia_Bacteroidales_*Bacteroidaceae_Bacteroides* | 0.331132722050617 | 0.753357491279219 | 0.995331457354929 |
| Bacteroidetes_Bacteroidia_Bacteroidales_*Porphyromonadaceae_Barnesiella* | 0.265833101715174 | 0.753357491279219 | 1.1300476177229 |
| Bacteroidetes_Bacteroidia_Bacteroidales_*Porphyromonadaceae_Macellibacteroides* | 0.348731220728654 | 0.753357491279219 | 1.6521483942384 |
| Bacteroidetes_Bacteroidia_Bacteroidales_*Porphyromonadaceae_Paludibacter* | 0.623968027230411 | 0.943351769816377 | 1.25692636034948 |

| | | | |
|---|---|---|---|
| Bacteroidetes_Bacteroidia_Bacteroidales_*Rikenellaceae_Alistipes* | 0.360733944717324 | 0.753357491279219 | 1.84643208487819 |
| Firmicutes_Bacilli_Bacillales_*Bacillaceae_Bacillus* | 0.882687544255599 | 0.943351769816377 | 0.954196854738033 |
| Firmicutes_Bacilli_Bacillales_*FamilyXIIncertaeSedis_Gemella* | 0.542700070626903 | 0.943351769816377 | 1.16076905828699 |
| Firmicutes_Bacilli_Bacillales_*Staphylococcaceae_Staphylococcus* | 0.21798764843239 | 0.753357491279219 | 1.24487773773262 |
| Firmicutes_Bacilli_Lactobacillales_*Carnobacteriaceae_Granulicatella* | 0.707889008195246 | 0.943351769816377 | 1.2439092989594 |
| Firmicutes_Bacilli_Lactobacillales_*Enterococcaceae_Enterococcus* | 0.101032042517163 | 0.631450265732266 | 1.76511916480728 |
| Firmicutes_Bacilli_Lactobacillales_*Lactobacillaceae_Lactobacillus* | 0.500973844121604 | 0.927729340965934 | 0.804214938267482 |
| Firmicutes_Bacilli_Lactobacillales_*Streptococcaceae_Streptococcus* | 0.651108190236675 | 0.943351769816377 | 1.07974333351745 |
| Firmicutes_Clostridia_Clostridiales_*Clostridiaceae_Clostridium* | 0.227826269520595 | 0.753357491279219 | 0.700917922377601 |
| Firmicutes_Clostridia_Clostridiales_*FamilyXIIncertaeSedis_Finegoldia* | 0.361611595814025 | 0.753357491279219 | 1.49846084367768 |
| Firmicutes_Clostridia_Clostridiales_*FamilyXIIncertaeSedis_Parvimonas* | 0.416004456722591 | 0.832008913445182 | 1.51746487230656 |
| Firmicutes_Clostridia_Clostridiales_*FamilyXIIncertaeSedis_Peptoniphilus* | 2.72141025767039e-05 | 0.000453568376278398 | 6.62715197878328 |
| Firmicutes_Clostridia_Clostridiales_*Lachnospiraceae_Anaerostipes* | 1.25631906411065e-07 | 3.14079766027662e-06 | 0.0981605518154673 |
| Firmicutes_Clostridia_Clostridiales_*Lachnospiraceae_Blautia* | 0.717895235082918 | 0.943351769816377 | 0.820745541984529 |
| Firmicutes_Clostridia_Clostridiales_*Lachnospiraceae_Coprococcus* | 0.0789920482891232 | 0.627865040235376 | 1.94302993200634 |
| Firmicutes_Clostridia_Clostridiales_*Lachnospiraceae_Dorea* | 0.943351769816377 | 0.943351769816377 | 0.950039186661183 |
| Firmicutes_Clostridia_Clostridiales_*Lachnospiraceae_IncertaeSedis* | 0.217003059274212 | 0.753357491279219 | 1.4938090127446 |
| Firmicutes_Clostridia_Clostridiales_*Lachnospiraceae_uncultured* | 0.920531438063263 | 0.943351769816377 | 1.04318888813244 |
| Firmicutes_Clostridia_Clostridiales_*Peptostreptococcaceae_IncertaeSedis* | 0.47875271709744 | 0.920678302110462 | 0.626926018449662 |
| Firmicutes_Clostridia_Clostridiales_*Ruminococcaceae_Faecalibacterium* | 9.06959254779924e-11 | 4.53479627389962e-09 | 4.71104212358456 |
| Firmicutes_Clostridia_Clostridiales_*Ruminococcaceae_IncertaeSedis* | 0.691210203466334 | 0.943351769816377 | 1.23852426765541 |
| Firmicutes_Clostridia_Clostridiales_*Ruminococcaceae_Subdoligranulum* | 0.799371396562985 | 0.943351769816377 | 1.10831249728546 |

| | | | |
|---|---|---|---|
| Firmicutes_Clostridia_Clostridiales_*Ruminococcaceae_uncultured* | 0.918608929690271 | 0.943351769816377 | 0.948712370708855 |
| Firmicutes_Erysipelotrichia_Erysipelotrichales_*Erysipelotrichaceae_Catenibacterium* | 0.799229385676864 | 0.943351769816377 | 0.93614407692264 |
| Firmicutes_Erysipelotrichia_Erysipelotrichales_*Erysipelotrichaceae_Solobacterium* | 0.861671594028878 | 0.943351769816377 | 0.956376106632675 |
| Firmicutes_Negativicutes_Selenomonadales_*Acidaminococcaceae_Acidaminococcus* | 0.178702375937834 | 0.753357491279219 | 2.32241185659108 |
| Firmicutes_Negativicutes_Selenomonadales_*Acidaminococcaceae_uncultured* | 0.115584164388514 | 0.642134246602855 | 2.43650818097107 |
| Firmicutes_Negativicutes_Selenomonadales_*Veillonellaceae_uncultured* | 0.941539996615444 | 0.943351769816377 | 1.05218549479273 |
| Firmicutes_Negativicutes_Selenomonadales_*Veillonellaceae_Veillonella* | 0.0879011056329527 | 0.627865040235376 | 0.967426789739684 |
| Proteobacteria_Betaproteobacteria_Burkholderiales_*Burkholderiaceae_Ralstonia* | 0.886899040315956 | 0.943351769816377 | 0.34094591385051 |
| Proteobacteria_Betaproteobacteria_Burkholderiales_*Oxalobacteraceae_Oxalobacter* | 0.782757791123717 | 0.943351769816377 | 0.800140187209349 |
| Proteobacteria_Deltaproteobacteria_Desulfovibrionales_*Desulfovibrionaceae_Desulfovibrio* | 0.774523182068567 | 0.943351769816377 | 0.821186926845137 |
| Proteobacteria_Gammaproteobacteria_Enterobacteriales_*Enterobacteriaceae_Citrobacter* | 0.580701351592434 | 0.943351769816377 | 0.877840271857179 |
| Proteobacteria_Gammaproteobacteria_Enterobacteriales_*Enterobacteriaceae_Enterobacter* | 0.620194554294082 | 0.943351769816377 | 0.889679730006872 |
| Proteobacteria_Gammaproteobacteria_Enterobacteriales_*Enterobacteriaceae_Klebsiella* | 0.263257363632005 | 0.753357491279219 | 1.45157821620229 |
| Proteobacteria_Gammaproteobacteria_Enterobacteriales_*Enterobacteriaceae_Proteus* | 0.00272552404838168 | 0.034069050604771 | 2.39994581082217 |
| Proteobacteria_Gammaproteobacteria_Enterobacteriales_*Enterobacteriaceae_Salmonella* | 0.743754288624893 | 0.943351769816377 | 0.907374040607519 |
| Proteobacteria_Gammaproteobacteria_Enterobacteriales_*Enterobacteriaceae_Serratia* | 0.0206789486452071 | 0.206789486452071 | 0.403025547853318 |
| Proteobacteria_Gammaproteobacteria_Pasteurellales_*Pasteurellaceae_Haemophilus* | 0.276088034702529 | 0.753357491279219 | 1.43281977324919 |
| Verrucomicrobia_Verrucomicrobiae_Verrucomicrobiales_*Verrucomicrobiaceae_Akkermansia* | 0.717326657274052 | 0.943351769816377 | 1.18153977701517 |

| **Supplemental Table S7. Microbiota differences in infants at family level.** P values for microbiota differences at six weeks of age between infants who developed a respiratory tract infection episode in the first three and six months of life and randomly selected infants remaining healthy | | | |
|---|---|---|---|
| **Taxon (Infection episode in the first 3 months)** | 1_p | 1_p_FDR | FoldChange_1 |
| Actinobacteria_Actinobacteria_Actinomycetales_*Actinomycetaceae* | 0.650474993252968 | 0.780023090873471 | 1.77496756035744 |
| Actinobacteria_Actinobacteria_Bifidobacteriales_*Bifidobacteriaceae* | 0.151800768984311 | 0.440222230054503 | 0.832309872509479 |
| Actinobacteria_Actinobacteria_Corynebacteriales_*Corynebacteriaceae* | 0.360231290946799 | 0.570667702622938 | 4.20249449659533 |
| Actinobacteria_Actinobacteria_Micrococcales_*Micrococcaceae* | 0.066675775029757 | 0.322266245977159 | 1.88415523588762 |
| Actinobacteria_Coriobacteriia_Coriobacteriales_*Coriobacteriaceae* | 0.391199705930494 | 0.570667702622938 | 1.8549135390735 |
| Bacteroidetes_Bacteroidia_Bacteroidales_*Bacteroidaceae* | 0.31160092354709 | 0.570667702622938 | 0.76522499886056 |
| Bacteroidetes_Bacteroidia_Bacteroidales_*Porphyromonadaceae* | 0.141386360541391 | 0.440222230054503 | 2.12232900215464 |
| Bacteroidetes_Bacteroidia_Bacteroidales_*Prevotellaceae* | 2.68635869022728e-07 | 7.79044020165912e-06 | 167.680895567543 |
| Bacteroidetes_Bacteroidia_Bacteroidales_*Rikenellaceae* | 0.0300991927221319 | 0.218219147235456 | 7.71935443290674 |
| Firmicutes_Bacilli_Bacillales_*Bacillaceae* | 0.220363299496603 | 0.532544640450125 | 1.60270511471178 |
| Firmicutes_Bacilli_Bacillales_*FamilyXIIncertaeSedis* | 0.992134149159371 | 0.992134149159371 | 0.987640015498889 |
| Firmicutes_Bacilli_Bacillales_*Staphylococcaceae* | 0.0927493087154563 | 0.336216244093529 | 1.60822054575943 |
| Firmicutes_Bacilli_Lactobacillales_*Enterococcaceae* | 0.0812220981544721 | 0.336216244093529 | 0.446758051476786 |
| Firmicutes_Bacilli_Lactobacillales_*Lactobacillaceae* | 0.672433699028855 | 0.780023090873471 | 1.17872745673855 |
| Firmicutes_Bacilli_Lactobacillales_*Streptococcaceae* | 0.375265206729065 | 0.570667702622938 | 0.999847826333183 |
| Firmicutes_Clostridia_Clostridiales_*Clostridiaceae* | 0.405783893679176 | 0.570667702622938 | 1.06843529019181 |
| Firmicutes_Clostridia_Clostridiales_FamilyXIIncertaeSedis | 0.376443901695363 | 0.570667702622938 | 1.54070808303379 |
| Firmicutes_Clostridia_Clostridiales_*Lachnospiraceae* | 0.840078752423294 | 0.870081565009841 | 1.07920349395694 |
| Firmicutes_Clostridia_Clostridiales_*Peptostreptococcaceae* | 0.215324408185059 | 0.532544640450125 | 2.75422487530468 |
| Firmicutes_Clostridia_Clostridiales_*Ruminococcaceae* | 0.00041986993564205 | 0.00405874271120649 | 3.41955805015177 |
| Firmicutes_Erysipelotrichia_Erysipelotrichales_*Erysipelotrichaceae* | 4.43653063039582e-05 | 0.000643296941407394 | 4.86686879146477 |
| Firmicutes_Negativicutes_Selenomonadales_*Acidaminococcaceae* | 0.333982982864085 | 0.570667702622938 | 1.9541864303007 |
| Firmicutes_Negativicutes_Selenomonadales_*Veillonellaceae* | 0.368087776768014 | 0.570667702622938 | 1.34570841650934 |
| Proteobacteria_Betaproteobacteria_Burkholderiales_*Burkholderiaceae* | 0.747930121612851 | 0.822924878918212 | 0.726461871870443 |
| Proteobacteria_Betaproteobacteria_Burkholderiales_*Oxalobacteraceae* | 0.766171438992818 | 0.822924878918212 | 0.739728633001375 |
| Proteobacteria_Deltaproteobacteria_Desulfovibrionales_*Desulfovibrionaceae* | 0.655113140189149 | 0.780023090873471 | 1.48322017785178 |
| Proteobacteria_Gammaproteobacteria_Enterobacteriales_*Enterobacteriaceae* | 0.427635860740561 | 0.570667702622938 | 1.16782356260172 |

| | | | |
|---|---|---|---|
| Proteobacteria_Gammaproteobacteria_Pasteurellales_*Pasteurellaceae* | 0.0536106931216654 | 0.31094202010566 | 0.410052585114908 |
| Verrucomicrobia_Verrucomicrobiae_Verrucomicrobiales_*Verrucomicrobiaceae* | 0.432920326127746 | 0.570667702622938 | 0.601052842495342 |
| **Taxon (Infection episode in the first 6 months)** | | | |
| Actinobacteria_Actinobacteria_Actinomycetales_*Actinomycetaceae* | 0.991314907359731 | 0.991314907359731 | 1.10720982880416 |
| Actinobacteria_Actinobacteria_Bifidobacteriales_*Bifidobacteriaceae* | 0.852040681833812 | 0.915154806414094 | 0.981618790383195 |
| Actinobacteria_Actinobacteria_Corynebacteriales_*Corynebacteriaceae* | 0.686980247659205 | 0.815654544774148 | 0.990489641359956 |
| Actinobacteria_Actinobacteria_Micrococcales_*Micrococcaceae* | 0.0950099920835892 | 0.463362392968158 | 0.667029285336955 |
| Actinobacteria_Coriobacteriia_Coriobacteriales_*Coriobacteriaceae* | 0.262048724088106 | 0.506627533237006 | 1.39751587299125 |
| Bacteroidetes_Bacteroidia_Bacteroidales_*Bacteroidaceae* | 0.908900200760383 | 0.941360922216111 | 0.983064341466551 |
| Bacteroidetes_Bacteroidia_Bacteroidales_*Porphyromonadaceae* | 0.139392508748451 | 0.463362392968158 | 1.74631084654477 |
| Bacteroidetes_Bacteroidia_Bacteroidales_*Prevotellaceae* | 0.188679229130479 | 0.465993082482256 | 2.63717576480505 |
| Bacteroidetes_Bacteroidia_Bacteroidales_*Rikenellaceae* | 0.132515406163256 | 0.463362392968158 | 2.89928152551422 |
| Firmicutes_Bacilli_Bacillales_*Bacillaceae* | 0.494099853872571 | 0.796049764572475 | 1.21874476287684 |
| Firmicutes_Bacilli_Bacillales_*FamilyXIIncertaeSedis* | 0.239925841492768 | 0.496989243092162 | 0.730570835001373 |
| Firmicutes_Bacilli_Bacillales_*Staphylococcaceae* | 0.0704847202778728 | 0.463362392968158 | 0.669807649050345 |
| Firmicutes_Bacilli_Lactobacillales_*Enterococcaceae* | 0.143802121955635 | 0.463362392968158 | 0.611408393072119 |
| Firmicutes_Bacilli_Lactobacillales_*Lactobacillaceae* | 0.424914249771488 | 0.72485372019842 | 0.797577418166728 |
| Firmicutes_Bacilli_Lactobacillales_*Streptococcaceae* | 0.109435621359497 | 0.463362392968158 | 0.930155846366546 |
| Firmicutes_Clostridia_Clostridiales_*Clostridiaceae* | 0.56563335521062 | 0.810522146056827 | 0.953842440017138 |
| Firmicutes_Clostridia_Clostridiales_*FamilyXIIncertaeSedis* | 0.192824723785761 | 0.465993082482256 | 1.55485727850246 |
| Firmicutes_Clostridia_Clostridiales_*Lachnospiraceae* | 0.230455202951373 | 0.496989243092162 | 1.38323302334928 |
| Firmicutes_Clostridia_Clostridiales_*Peptostreptococcaceae* | 0.348873772994425 | 0.632333713552395 | 0.56796241131015 |
| Firmicutes_Clostridia_Clostridiales_*Ruminococcaceae* | 0.139728564914104 | 0.463362392968158 | 1.44831203520162 |
| Firmicutes_Erysipelotrichia_Erysipelotrichales_*Erysipelotrichaceae* | 0.703150469632887 | 0.815654544774148 | 1.11207933883793 |
| Firmicutes_Negativicutes_Selenomonadales_*Acidaminococcaceae* | 0.0699233967137517 | 0.463362392968158 | 2.37251662580397 |
| Firmicutes_Negativicutes_Selenomonadales_*Veillonellaceae* | 0.633521417482696 | 0.810522146056827 | 0.896602371717262 |
| Proteobacteria_Betaproteobacteria_Burkholderiales_*Burkholderiaceae* | 0.642827908941622 | 0.810522146056827 | 0.719393106764263 |
| Proteobacteria_Betaproteobacteria_Burkholderiales_*Oxalobacteraceae* | 0.534478673564412 | 0.810522146056827 | 0.650364577335011 |
| Proteobacteria_Deltaproteobacteria_Desulfovibrionales_*Desulfovibrionaceae* | 0.167821190752909 | 0.465993082482256 | 2.39157242186895 |
| Proteobacteria_Gammaproteobacteria_Enterobacteriales_*Enterobacteriaceae* | 0.755549079685816 | 0.842727819649564 | 1.02648355969294 |

| | | | |
|---|---|---|---|
| Proteobacteria_Gammaproteobacteria_Pasteurellales_*Pasteurellaceae* | 0.0288157910289914 | 0.463362392968158 | 0.492058581311938 |
| Verrucomicrobia_Verrucomicrobiae_Verrucomicrobiales_*Verrucomicrobiaceae* | 0.614991790813012 | 0.810522146056827 | 0.791500224626292 |

**Supplemental Table S8. Microbiota differences in infants at genus level.** P values for microbiota differences at six weeks of age between infants who developed a respiratory tract infection episode in the first three and six months of life and randomly selected infants remaining healthy

| **Taxon (Infection episode in the first 3 months)** | 1_p | 1_p_FDR | FoldChange_1 |
|---|---|---|---|
| Actinobacteria_Actinobacteria_Actinomycetales_*Actinomycetaceae_Actinomyces* | 0.650474993252968 | 0.769045860222044 | 1.77496756035744 |
| Actinobacteria_Actinobacteria_Bifidobacteriales_*Bifidobacteriaceae_Bifidobacterium* | 0.151800768984311 | 0.330277996812226 | 0.832309872509479 |
| Actinobacteria_Actinobacteria_Corynebacteriales_*Corynebacteriaceae_Corynebacterium* | 0.360231290946799 | 0.589469385185671 | 4.20249449659533 |
| Actinobacteria_Actinobacteria_Micrococcales_*Micrococcaceae_Rothia* | 0.066675775029757 | 0.248692077403514 | 1.88415523588762 |
| Actinobacteria_Coriobacteriia_Coriobacteriales_*Coriobacteriaceae_Atopobium* | 0.012563247696797 | 0.0969164822324337 | 9.51733524624596 |
| Actinobacteria_Coriobacteriia_Coriobacteriales_*Coriobacteriaceae_Collinsella* | 0.155009469344253 | 0.330277996812226 | 1.83309825155534 |
| Actinobacteria_Coriobacteriia_Coriobacteriales_*Coriobacteriaceae_Slackia* | 0.341136102653672 | 0.575667173228072 | 1.63908540796439 |
| Actinobacteria_Coriobacteriia_Coriobacteriales_*Coriobacteriaceae_uncultured* | 0.06653251227106 | 0.248692077403514 | 2.43949078801386 |
| Bacteroidetes_Bacteroidia_Bacteroidales_*Bacteroidaceae_Bacteroides* | 0.31160092354709 | 0.542788705533641 | 0.76522499886056 |
| Bacteroidetes_Bacteroidia_Bacteroidales_*Porphyromonadaceae_Barnesiella* | 0.453829626897866 | 0.612669996312119 | 1.48731043224107 |
| Bacteroidetes_Bacteroidia_Bacteroidales_*Porphyromonadaceae_Macellibacteroides* | 2.40168731669873e-05 | 0.000440911786014165 | 23.0367083136653 |
| Bacteroidetes_Bacteroidia_Bacteroidales_*Porphyromonadaceae_Paludibacter* | 0.159050803010225 | 0.330277996812226 | 2.30997263465345 |
| Bacteroidetes_Bacteroidia_Bacteroidales_*Prevotellaceae_Prevotella* | 3.26601322973455e-05 | 0.000440911786014165 | 68.5581584569002 |
| Bacteroidetes_Bacteroidia_Bacteroidales_*Rikenellaceae_Alistipes* | 0.0300991927221319 | 0.147759673363193 | 7.71935443290674 |
| Firmicutes_Bacilli_Bacillales_*Bacillaceae_Bacillus* | 0.220363299496603 | 0.410331661131606 | 1.60270511471178 |
| Firmicutes_Bacilli_Bacillales_*FamilyXIIncertaeSedis_Gemella* | 0.992134149159371 | 0.992134149159371 | 0.987640015498889 |
| Firmicutes_Bacilli_Bacillales_*Staphylococcaceae_Staphylococcus* | 0.0927493087154563 | 0.253128156695232 | 1.60822054575943 |
| Firmicutes_Bacilli_Lactobacillales_*Enterococcaceae_Enterococcus* | 0.0812220981544721 | 0.253128156695232 | 0.446758051476786 |
| Firmicutes_Bacilli_Lactobacillales_*Lactobacillaceae_Lactobacillus* | 0.672433699028855 | 0.772583398884216 | 1.17872745673855 |
| Firmicutes_Bacilli_Lactobacillales_*Streptococcaceae_Streptococcus* | 0.375265206729065 | 0.591157512146244 | 0.999847826333183 |
| Firmicutes_Clostridia_Clostridiales_*Clostridiaceae_Clostridium* | 0.405783893679176 | 0.599428143869187 | 1.06843529019181 |

| | | | |
|---|---|---|---|
| Firmicutes_Clostridia_Clostridiales_*FamilyXIIncertaeSedis_Finegoldia* | 0.954212683960934 | 0.972216696865858 | 0.979589233906521 |
| Firmicutes_Clostridia_Clostridiales_*FamilyXIIncertaeSedis_Peptoniphilus* | 0.0188546703736295 | 0.110644521445833 | 3.76611544530523 |
| Firmicutes_Clostridia_Clostridiales_*Lachnospiraceae_Anaerostipes* | 2.90377110630415e-06 | 0.000156803639740424 | 0.0340844789445113 |
| Firmicutes_Clostridia_Clostridiales_*Lachnospiraceae_Blautia* | 0.0157019187284476 | 0.105987951417021 | 0.289317882776823 |
| Firmicutes_Clostridia_Clostridiales_*Lachnospiraceae_Coprococcus* | 0.552560367621446 | 0.67930842911734 | 1.35893129056515 |
| Firmicutes_Clostridia_Clostridiales_*Lachnospiraceae_Dorea* | 0.849649720325466 | 0.902572197818362 | 1.06973059065869 |
| Firmicutes_Clostridia_Clostridiales_*Lachnospiraceae_IncertaeSedis* | 0.533148584684211 | 0.67930842911734 | 1.33504400898353 |
| Firmicutes_Clostridia_Clostridiales_*Lachnospiraceae_Pseudobutyrivibrio* | 0.061867503443364 | 0.248692077403514 | 2.12606981758782 |
| Firmicutes_Clostridia_Clostridiales_*Lachnospiraceae_Roseburia* | 0.0937511691463822 | 0.253128156695232 | 4.42052942861209 |
| Firmicutes_Clostridia_Clostridiales_*Lachnospiraceae_uncultured* | 0.287197587070771 | 0.516955656727388 | 1.77410078767708 |
| Firmicutes_Clostridia_Clostridiales_*Peptostreptococcaceae_IncertaeSedis* | 0.215324408185059 | 0.410331661131606 | 2.75422487530468 |
| Firmicutes_Clostridia_Clostridiales_*Ruminococcaceae_Faecalibacterium* | 0.00306340054313806 | 0.0275706048882426 | 2.75704235498947 |
| Firmicutes_Clostridia_Clostridiales_*Ruminococcaceae_IncertaeSedis* | 0.944129700218503 | 0.972216696865858 | 1.0457040336543 |
| Firmicutes_Clostridia_Clostridiales_*Ruminococcaceae_Subdoligranulum* | 0.000152341615852482 | 0.0016452894512068 | 6.37185369552076 |
| Firmicutes_Clostridia_Clostridiales_*Ruminococcaceae_uncultured* | 0.086758557528722 | 0.253128156695232 | 2.95676928293298 |
| Firmicutes_Erysipelotrichia_Erysipelotrichales_*Erysipelotrichaceae_Catenibacterium* | 3.14668095409256e-05 | 0.000440911786014165 | 4.78888696264848 |
| Firmicutes_Erysipelotrichia_Erysipelotrichales_*Erysipelotrichaceae_Solobacterium* | 0.090423646489175 | 0.253128156695232 | 5.26274657856834 |
| Firmicutes_Negativicutes_Selenomonadales_*Acidaminococcaceae_Acidaminococcus* | 0.431959591645793 | 0.599428143869187 | 1.91946684384199 |
| Firmicutes_Negativicutes_Selenomonadales_*Acidaminococcaceae_uncultured* | 0.165138998406113 | 0.330277996812226 | 2.81806532654246 |
| Firmicutes_Negativicutes_Selenomonadales_*Veillonellaceae_Megasphaera* | 0.481575039803354 | 0.634269564619052 | 0.410037233021345 |
| Firmicutes_Negativicutes_Selenomonadales_*Veillonellaceae_Selenomonas* | 0.852429297939564 | 0.902572197818362 | 1.21322967904745 |
| Firmicutes_Negativicutes_Selenomonadales_*Veillonellaceae_Veillonella* | 0.383157646761454 | 0.591157512146244 | 1.3440187683809 |
| Proteobacteria_Betaproteobacteria_Burkholderiales_*Burkholderiaceae_Ralstonia* | 0.747930121612851 | 0.841421386814457 | 0.726461871870443 |

| | | | |
|---|---|---|---|
| Proteobacteria_Betaproteobacteria_Burkholderiales_*Oxalobacteraceae_Oxalobacter* | 0.766171438992818 | 0.844352198073718 | 0.739728633001375 |
| Proteobacteria_Deltaproteobacteria_Desulfovibrionales_*Desulfovibrionaceae_Desulfovibrio* | 0.655113140189149 | 0.769045860222044 | 1.48322017785178 |
| Proteobacteria_Gammaproteobacteria_Enterobacteriales_*Enterobacteriaceae_Citrobacter* | 0.422702987674559 | 0.599428143869187 | 1.00160204250729 |
| Proteobacteria_Gammaproteobacteria_Enterobacteriales_*Enterobacteriaceae_Enterobacter* | 0.164578115279768 | 0.330277996812226 | 1.20599868466928 |
| Proteobacteria_Gammaproteobacteria_Enterobacteriales_*Enterobacteriaceae_Klebsiella* | 0.139217341050693 | 0.330277996812226 | 2.04090112714748 |
| Proteobacteria_Gammaproteobacteria_Enterobacteriales_*Enterobacteriaceae_Proteus* | 0.14470784766009 | 0.330277996812226 | 0.507970484531685 |
| Proteobacteria_Gammaproteobacteria_Enterobacteriales_*Enterobacteriaceae_Salmonella* | 0.0204897261936728 | 0.110644521445833 | 2.49948559928508 |
| Proteobacteria_Gammaproteobacteria_Enterobacteriales_*Enterobacteriaceae_Serratia* | 0.553510571873388 | 0.67930842911734 | 1.34843852697919 |
| Proteobacteria_Gammaproteobacteria_Pasteurellales_*Pasteurellaceae_Haemophilus* | 0.0690811326120871 | 0.248692077403514 | 0.430150738867734 |
| Verrucomicrobia_Verrucomicrobiae_Verrucomicrobiales_*Verrucomicrobiaceae_Akkermansia* | 0.432920326127746 | 0.599428143869187 | 0.601052842495342 |
| **Taxon (Infection episode in the first 6 months)** | | | |
| Actinobacteria_Actinobacteria_Actinomycetales_*Actinomycetaceae_Actinomyces* | 0.991314907359731 | 0.991314907359731 | 1.10720982880416 |
| Actinobacteria_Actinobacteria_Bifidobacteriales_*Bifidobacteriaceae_Bifidobacterium* | 0.852040681833812 | 0.926049261152089 | 0.981618790383195 |
| Actinobacteria_Actinobacteria_Corynebacteriales_*Corynebacteriaceae_Corynebacterium* | 0.686980247659205 | 0.850145652923981 | 0.990489641359956 |
| Actinobacteria_Actinobacteria_Micrococcales_*Micrococcaceae_Rothia* | 0.0950099920835892 | 0.383284881788365 | 0.667029285336955 |
| Actinobacteria_Coriobacteriia_Coriobacteriales_*Coriobacteriaceae_Atopobium* | 0.299046958365058 | 0.645941430068525 | 1.80003248811076 |
| Actinobacteria_Coriobacteriia_Coriobacteriales_*Coriobacteriaceae_Collinsella* | 0.443412761071995 | 0.772396422512507 | 1.32986705580502 |
| Actinobacteria_Coriobacteriia_Coriobacteriales_*Coriobacteriaceae_Slackia* | 0.328254891427217 | 0.681760159118066 | 1.43221441421617 |
| Actinobacteria_Coriobacteriia_Coriobacteriales_*Coriobacteriaceae_uncultured* | 0.0586345623250147 | 0.383284881788365 | 1.93523486720249 |

| | | | |
|---|---|---|---|
| Bacteroidetes_Bacteroidia_Bacteroidales_*Bacteroidaceae_Bacteroides* | 0.908900200760383 | 0.926049261152089 | 0.983064341466551 |
| Bacteroidetes_Bacteroidia_Bacteroidales_*Porphyromonadaceae_Barnesiella* | 0.501445967458897 | 0.788433652931108 | 1.30168539602491 |
| Bacteroidetes_Bacteroidia_Bacteroidales_*Porphyromonadaceae_Macellibacteroides* | 0.00875356299026547 | 0.118173100368584 | 4.07203364268613 |
| Bacteroidetes_Bacteroidia_Bacteroidales_*Porphyromonadaceae_Paludibacter* | 0.291005592867643 | 0.645941430068525 | 1.60273259223596 |
| Bacteroidetes_Bacteroidia_Bacteroidales_*Prevotellaceae_Prevotella* | 0.113565890900256 | 0.383284881788365 | 3.09553915133088 |
| Bacteroidetes_Bacteroidia_Bacteroidales_*Rikenellaceae_Alistipes* | 0.132515406163256 | 0.420931290165636 | 2.89928152551422 |
| Firmicutes_Bacilli_Bacillales_*Bacillaceae_Bacillus* | 0.494099853872571 | 0.788433652931108 | 1.21874476287684 |
| Firmicutes_Bacilli_Bacillales_*FamilyXIIncertaeSedis_Gemella* | 0.239925841492768 | 0.588908883664067 | 0.730570835001373 |
| Firmicutes_Bacilli_Bacillales_*Staphylococcaceae_Staphylococcus* | 0.0704847202778728 | 0.383284881788365 | 0.669807649050345 |
| Firmicutes_Bacilli_Lactobacillales_*Enterococcaceae_Enterococcus* | 0.143802121955635 | 0.431406365866906 | 0.611408393072119 |
| Firmicutes_Bacilli_Lactobacillales_*Lactobacillaceae_Lactobacillus* | 0.424914249771488 | 0.772396422512507 | 0.797577418166728 |
| Firmicutes_Bacilli_Lactobacillales_*Streptococcaceae_Streptococcus* | 0.109435621359497 | 0.383284881788365 | 0.930155846366546 |
| Firmicutes_Clostridia_Clostridiales_*Clostridiaceae_Clostridium* | 0.56563335521062 | 0.803794767930881 | 0.953842440017138 |
| Firmicutes_Clostridia_Clostridiales_*FamilyXIIncertaeSedis_Finegoldia* | 0.104285527945828 | 0.383284881788365 | 2.01482436140905 |
| Firmicutes_Clostridia_Clostridiales_*FamilyXIIncertaeSedis_Peptoniphilus* | 0.509009324132236 | 0.788433652931108 | 1.31821636237902 |
| Firmicutes_Clostridia_Clostridiales_*Lachnospiraceae_Anaerostipes* | 1.41871306768209e-08 | 7.6610505654833e-07 | 0.0461849713008327 |
| Firmicutes_Clostridia_Clostridiales_*Lachnospiraceae_Blautia* | 0.00344370754891436 | 0.0929801038206878 | 0.341912143680696 |
| Firmicutes_Clostridia_Clostridiales_*Lachnospiraceae_Coprococcus* | 0.368221039703723 | 0.710140576571466 | 1.39443456899718 |
| Firmicutes_Clostridia_Clostridiales_*Lachnospiraceae_Dorea* | 0.878839453698453 | 0.926049261152089 | 1.035506558302 |
| Firmicutes_Clostridia_Clostridiales_*Lachnospiraceae_IncertaeSedis* | 0.0851536387617155 | 0.383284881788365 | 1.7636051768724 |
| Firmicutes_Clostridia_Clostridiales_*Lachnospiraceae_Pseudobutyrivibrio* | 0.187706010477727 | 0.506806228289863 | 0.666110218336908 |
| Firmicutes_Clostridia_Clostridiales_*Lachnospiraceae_Roseburia* | 0.771286586660487 | 0.886239813672404 | 1.20376238596335 |

| | | | |
|---|---|---|---|
| Firmicutes_Clostridia_Clostridiales_*Lachnospiraceae_uncultured* | 0.631193198484134 | 0.826493025782085 | 0.823772374472428 |
| Firmicutes_Clostridia_Clostridiales_*Peptostreptococcaceae_IncertaeSedis* | 0.348873772994425 | 0.697747545988849 | 0.56796241131015 |
| Firmicutes_Clostridia_Clostridiales_*Ruminococcaceae_Faecalibacterium* | 0.432767116482171 | 0.772396422512507 | 0.820983717465659 |
| Firmicutes_Clostridia_Clostridiales_*Ruminococcaceae_IncertaeSedis* | 0.211856430080808 | 0.544773677350648 | 1.23794389327732 |
| Firmicutes_Clostridia_Clostridiales_*Ruminococcaceae_Subdoligranulum* | 0.0456039104446807 | 0.351801594858965 | 2.07616222116449 |
| Firmicutes_Clostridia_Clostridiales_*Ruminococcaceae_uncultured* | 0.513677282736724 | 0.788433652931108 | 1.34230702967903 |
| Firmicutes_Erysipelotrichia_Erysipelotrichales_*Erysipelotrichaceae_Catenibacterium* | 0.708669263931056 | 0.850403116717268 | 1.10606999860512 |
| Firmicutes_Erysipelotrichia_Erysipelotrichales_*Erysipelotrichaceae_Solobacterium* | 0.832633212610753 | 0.926049261152089 | 1.15131183619065 |
| Firmicutes_Negativicutes_Selenomonadales_*Acidaminococcaceae_Acidaminococcus* | 0.0924999166931523 | 0.383284881788365 | 2.48647995516522 |
| Firmicutes_Negativicutes_Selenomonadales_*Acidaminococcaceae_uncultured* | 0.0432508211181889 | 0.351801594858965 | 2.93005485179104 |
| Firmicutes_Negativicutes_Selenomonadales_*Veillonellaceae_Megasphaera* | 0.692711272752874 | 0.850145652923981 | 0.735101895128369 |
| Firmicutes_Negativicutes_Selenomonadales_*Veillonellaceae_Selenomonas* | 0.907513832123708 | 0.926049261152089 | 1.08104525566792 |
| Firmicutes_Negativicutes_Selenomonadales_*Veillonellaceae_Veillonella* | 0.605349549553824 | 0.826493025782085 | 0.886345363541012 |
| Proteobacteria_Betaproteobacteria_Burkholderiales_*Burkholderiaceae_Ralstonia* | 0.642827908941622 | 0.826493025782085 | 0.719393106764263 |
| Proteobacteria_Betaproteobacteria_Burkholderiales_*Oxalobacteraceae_Oxalobacter* | 0.534478673564412 | 0.788433652931108 | 0.650364577335011 |
| Proteobacteria_Deltaproteobacteria_Desulfovibrionales_*Desulfovibrionaceae_Desulfovibrio* | 0.167821190752909 | 0.476965489508268 | 2.39157242186895 |
| Proteobacteria_Gammaproteobacteria_Enterobacteriales_*Enterobacteriaceae_Citrobacter* | 0.771356874863018 | 0.886239813672404 | 1.05371576822415 |
| Proteobacteria_Gammaproteobacteria_Enterobacteriales_*Enterobacteriaceae_Enterobacter* | 0.878769268319209 | 0.926049261152089 | 0.97770555904067 |
| Proteobacteria_Gammaproteobacteria_Enterobacteriales_*Enterobacteriaceae_Klebsiella* | 0.540223058489833 | 0.788433652931108 | 1.20418626245985 |
| Proteobacteria_Gammaproteobacteria_Enterobacteriales_*Enterobacteriaceae_Proteus* | 0.282365390916059 | 0.645941430068525 | 1.27864119243251 |

| | | | |
|---|---|---|---|
| Proteobacteria_Gammaproteobacteria_Enterobacteriales_*Enterobacteriaceae_Salmonella* | 0.0083800365613389 | 0.118173100368584 | 2.07008486185199 |
| Proteobacteria_Gammaproteobacteria_Enterobacteriales_*Enterobacteriaceae_Serratia* | 0.0910215452084453 | 0.383284881788365 | 1.83533436096693 |
| Proteobacteria_Gammaproteobacteria_Pasteurellales_*Pasteurellaceae_Haemophilus* | 0.0351916971231286 | 0.351801594858965 | 0.503145111523451 |
| Verrucomicrobia_Verrucomicrobiae_Verrucomicrobiales_*Verrucomicrobiaceae_Akkermansia* | 0.614991790813012 | 0.826493025782085 | 0.791500224626292 |

| **Supplemental Table S9**. **Microbiota differences in infants at family level.** P values for microbiota differences at three weeks of age between infants who developed a respiratory tract infection episode in the first three and six months of life with the exclusion of infants with any infection before the first fecal sample and matched infants remaining healthy. | | | |
|---|---|---|---|
| **Taxon (Infection episode in the first 3 months)** | 1_p | 1_p_FDR | FoldChange_1 |
| Actinobacteria_Actinobacteria_Actinomycetales_*Actinomycetaceae* | 0.0334459899907411 | 0.217398934939817 | 5.57352474873554 |
| Actinobacteria_Actinobacteria_Bifidobacteriales_*Bifidobacteriaceae* | 0.758806122506926 | 0.948792793621264 | 0.928385284345777 |
| Actinobacteria_Actinobacteria_Micrococcales_*Micrococcaceae* | 0.0118221273844373 | 0.106490102245691 | 4.14008613601115 |
| Actinobacteria_Coriobacteriia_Coriobacteriales_*Coriobacteriaceae* | 0.505776812221687 | 0.948792793621264 | 1.07992182446594 |
| Bacteroidetes_Bacteroidia_Bacteroidales_*Bacteroidaceae* | 0.979254852392886 | 0.979254852392886 | 1.01370126822105 |
| Bacteroidetes_Bacteroidia_Bacteroidales_*Porphyromonadaceae* | 0.665451170266173 | 0.948792793621264 | 0.695542793115475 |
| Bacteroidetes_Bacteroidia_Bacteroidales_*Rikenellaceae* | 0.408789986881849 | 0.948792793621264 | 3.72932459940809 |
| Firmicutes_Bacilli_Bacillales_*Bacillaceae* | 0.71452681575764 | 0.948792793621264 | 1.31644135067721 |
| Firmicutes_Bacilli_Bacillales_*FamilyXIIncertaeSedis* | 0.655643197519158 | 0.948792793621264 | 0.810032838779865 |
| Firmicutes_Bacilli_Bacillales_*Staphylococcaceae* | 0.700828625219239 | 0.948792793621264 | 0.849962894954465 |
| Firmicutes_Bacilli_Lactobacillales_*Carnobacteriaceae* | 0.671750528693406 | 0.948792793621264 | 0.682617759724366 |
| Firmicutes_Bacilli_Lactobacillales_*Enterococcaceae* | 0.453742856864133 | 0.948792793621264 | 0.535924483123025 |
| Firmicutes_Bacilli_Lactobacillales_*Lactobacillaceae* | 0.859529014753091 | 0.948792793621264 | 1.1360101766855 |
| Firmicutes_Bacilli_Lactobacillales_*Streptococcaceae* | 0.153014961683632 | 0.663064833962404 | 0.57638720082601 |
| Firmicutes_Clostridia_Clostridiales_*Clostridiaceae* | 0.445508114406236 | 0.948792793621264 | 0.606629945425522 |
| Firmicutes_Clostridia_Clostridiales_*FamilyXIIncertaeSedis* | 0.638000683247989 | 0.948792793621264 | 1.60045839256158 |
| Firmicutes_Clostridia_Clostridiales_*Lachnospiraceae* | 0.850848709073336 | 0.948792793621264 | 0.882181187470113 |
| Firmicutes_Clostridia_Clostridiales_*Peptostreptococcaceae* | 0.875808732573474 | 0.948792793621264 | 1.21643480957773 |
| Firmicutes_Clostridia_Clostridiales_*Ruminococcaceae* | 0.463734144070816 | 0.948792793621264 | 1.62776519143658 |
| Firmicutes_Erysipelotrichia_Erysipelotrichales_*Erysipelotrichaceae* | 0.0122873194898875 | 0.106490102245691 | 40.19003477383 |
| Firmicutes_Negativicutes_Selenomonadales_*Acidaminococcaceae* | 0.830110973660231 | 0.948792793621264 | 1.15743370274969 |
| Firmicutes_Negativicutes_Selenomonadales_*Veillonellaceae* | 0.261527623265144 | 0.948792793621264 | 1.81199233829216 |
| Proteobacteria_Deltaproteobacteria_Desulfovibrionales_*Desulfovibrionaceae* | 0.351115311961038 | 0.948792793621264 | 1.83855011686025 |
| Proteobacteria_Gammaproteobacteria_Enterobacteriales_*Enterobacteriaceae* | 0.926470272297257 | 0.963529083189147 | 1.02067357762985 |
| Proteobacteria_Gammaproteobacteria_Pasteurellales_*Pasteurellaceae* | 0.0910070568494098 | 0.473236695616931 | 0.279842898321341 |
| Verrucomicrobia_Verrucomicrobiae_Verrucomicrobiales_Verrucomicrobiaceae | 0.00662977324464491 | 0.106490102245691 | 0.213023367084797 |

| Taxon (Infection episode in the first 6 months) | | | |
|---|---|---|---|
| Actinobacteria_Actinobacteria_Actinomycetales_*Actinomycetaceae* | 0.647081047077153 | 0.844319822460795 | 1.18108591591995 |
| Actinobacteria_Actinobacteria_Bifidobacteriales_*Bifidobacteriaceae* | 0.208700319384811 | 0.609780295419192 | 1.15661825379784 |
| Actinobacteria_Actinobacteria_Micrococcales_*Micrococcaceae* | 0.480752896420976 | 0.77258201704177 | 0.828904093683049 |
| Actinobacteria_Coriobacteriia_Coriobacteriales_*Coriobacteriaceae* | 0.806886358223346 | 0.844319822460795 | 1.27963596295677 |
| Bacteroidetes_Bacteroidia_Bacteroidales_*Bacteroidaceae* | 0.674021951979439 | 0.844319822460795 | 0.982692072362519 |
| Bacteroidetes_Bacteroidia_Bacteroidales_*Porphyromonadaceae* | 0.844319822460795 | 0.844319822460795 | 0.926525808717605 |
| Bacteroidetes_Bacteroidia_Bacteroidales_*Rikenellaceae* | 0.58206751321655 | 0.840764185757238 | 0.658632934541159 |
| Firmicutes_Bacilli_Bacillales_*Bacillaceae* | 0.766654199689884 | 0.844319822460795 | 0.901580270002105 |
| Firmicutes_Bacilli_Bacillales_*FamilyXIIncertaeSedis* | 0.79160555697773 | 0.844319822460795 | 0.940806608559011 |
| Firmicutes_Bacilli_Bacillales_*Staphylococcaceae* | 0.425143176775629 | 0.736914839744424 | 1.16466615162787 |
| Firmicutes_Bacilli_Lactobacillales_*Carnobacteriaceae* | 0.794686644942205 | 0.844319822460795 | 0.914722558925883 |
| Firmicutes_Bacilli_Lactobacillales_*Enterococcaceae* | 0.211077794568182 | 0.609780295419192 | 1.56102017753302 |
| Firmicutes_Bacilli_Lactobacillales_*Lactobacillaceae* | 0.505149780373465 | 0.77258201704177 | 0.794181005616416 |
| Firmicutes_Bacilli_Lactobacillales_*Streptococcaceae* | 0.82297518716924 | 0.844319822460795 | 1.04039095331817 |
| Firmicutes_Clostridia_Clostridiales_*Clostridiaceae* | 0.422855131179287 | 0.736914839744424 | 0.780082367225292 |
| Firmicutes_Clostridia_Clostridiales_*FamilyXIIncertaeSedis* | 0.194914804500813 | 0.609780295419192 | 1.74067420081654 |
| Firmicutes_Clostridia_Clostridiales_*Lachnospiraceae* | 0.148571032075768 | 0.609780295419192 | 1.51220138424187 |
| Firmicutes_Clostridia_Clostridiales_*Peptostreptococcaceae* | 0.103394995670941 | 0.537653977488893 | 0.372095238890143 |
| Firmicutes_Clostridia_Clostridiales_*Ruminococcaceae* | 0.400779614341007 | 0.736914839744424 | 1.26816891851422 |
| Firmicutes_Erysipelotrichia_Erysipelotrichales_*Erysipelotrichaceae* | 0.0851266048771703 | 0.537653977488893 | 0.599375660703688 |
| Firmicutes_Negativicutes_Selenomonadales_*Acidaminococcaceae* | 8.53771041245974e-05 | 0.00221980470723953 | 3.17474802826507 |
| Firmicutes_Negativicutes_Selenomonadales_*Veillonellaceae* | 0.00695928822985125 | 0.0904707469880663 | 0.89764395683087 |
| Proteobacteria_Deltaproteobacteria_Desulfovibrionales_*Desulfovibrionaceae* | 0.325176695373587 | 0.736914839744424 | 2.13380778023727 |
| Proteobacteria_Gammaproteobacteria_Enterobacteriales_*Enterobacteriaceae* | 0.328569039838778 | 0.736914839744424 | 0.911894274423408 |
| Proteobacteria_Gammaproteobacteria_Pasteurellales_*Pasteurellaceae* | 0.38534539797213 | 0.736914839744424 | 0.754393418200599 |
| Verrucomicrobia_Verrucomicrobiae_Verrucomicrobiales_*Verrucomicrobiaceae* | 0.0618481388774566 | 0.536017203604624 | 0.366217766481984 |

**Supplemental Table S10**. **Microbiota differences in infants at genus level.** P values for microbiota differences at three weeks of age between infants who developed a respiratory tract infection episode in the first three and six months of life with the exclusion of infants with any infection before the first fecal sample and matched infants remaining healthy.

| **Taxon (Infection episode in the first 3 months)** | 1_p | 1_p_FDR | FoldChange_1 |
|---|---|---|---|
| Actinobacteria_Actinobacteria_Actinomycetales_*Actinomycetaceae_Actinomyces* | 0.0334459899907411 | 0.133783959962964 | 5.57352474873554 |
| Actinobacteria_Actinobacteria_Bifidobacteriales_*Bifidobacteriaceae_Bifidobacterium* | 0.758806122506926 | 0.910350471736992 | 0.928385284345777 |
| Actinobacteria_Actinobacteria_Micrococcales_*Micrococcaceae_Rothia* | 0.0118221273844373 | 0.0614750623990739 | 4.14008613601115 |
| Actinobacteria_Coriobacteriia_Coriobacteriales_*Coriobacteriaceae_Collinsella* | 0.499351244389395 | 0.910350471736992 | 1.33722718408185 |
| Actinobacteria_Coriobacteriia_Coriobacteriales_*Coriobacteriaceae_Slackia* | 0.69995483432377 | 0.910350471736992 | 0.758851028772859 |
| Actinobacteria_Coriobacteriia_Coriobacteriales_*Coriobacteriaceae_uncultured* | 0.666992844435204 | 0.910350471736992 | 0.571997281320821 |
| Bacteroidetes_Bacteroidia_Bacteroidales_*Bacteroidaceae_Bacteroides* | 0.979254852392886 | 0.998455927930001 | 1.01370126822105 |
| Bacteroidetes_Bacteroidia_Bacteroidales_*Porphyromonadaceae_Barnesiella* | 0.770296553008224 | 0.910350471736992 | 0.761941039798369 |
| Bacteroidetes_Bacteroidia_Bacteroidales_*Porphyromonadaceae_Macellibacteroides* | 0.313259058184568 | 0.819390998986889 | 2.9714787200677 |
| Bacteroidetes_Bacteroidia_Bacteroidales_*Porphyromonadaceae_Paludibacter* | 0.663853258916626 | 0.910350471736992 | 0.672020030008064 |
| Bacteroidetes_Bacteroidia_Bacteroidales_*Rikenellaceae_Alistipes* | 0.408789986881849 | 0.907485713728266 | 3.72932459940809 |
| Firmicutes_Bacilli_Bacillales_*Bacillaceae_Bacillus* | 0.71452681575764 | 0.910350471736992 | 1.31644135067721 |
| Firmicutes_Bacilli_Bacillales_*FamilyXIIncertaeSedis_Gemella* | 0.655643197519158 | 0.910350471736992 | 0.810032838779865 |
| Firmicutes_Bacilli_Bacillales_*Staphylococcaceae_Staphylococcus* | 0.700828625219239 | 0.910350471736992 | 0.849962894954465 |
| Firmicutes_Bacilli_Lactobacillales_*Carnobacteriaceae_Granulicatella* | 0.671750528693406 | 0.910350471736992 | 0.682617759724366 |
| Firmicutes_Bacilli_Lactobacillales_*Enterococcaceae_Enterococcus* | 0.453742856864133 | 0.907485713728266 | 0.535924483123025 |
| Firmicutes_Bacilli_Lactobacillales_*Lactobacillaceae_Lactobacillus* | 0.859529014753091 | 0.948792793621264 | 1.1360101766855 |
| Firmicutes_Bacilli_Lactobacillales_*Streptococcaceae_Streptococcus* | 0.153014961683632 | 0.468045765149932 | 0.57638720082601 |

| | | | |
|---|---|---|---|
| Firmicutes_Clostridia_Clostridiales_*Clostridiaceae_Clostridium* | 0.445508114406236 | 0.907485713728266 | 0.606629945425522 |
| Firmicutes_Clostridia_Clostridiales_*FamilyXIIncertaeSedis_Finegoldia* | 0.866751686122848 | 0.948792793621264 | 1.21049195544314 |
| Firmicutes_Clostridia_Clostridiales_*FamilyXIIncertaeSedis_Parvimonas* | 0.840260952358951 | 0.948792793621264 | 1.29013709555587 |
| Firmicutes_Clostridia_Clostridiales_*FamilyXIIncertaeSedis_Peptoniphilus* | 0.180796978169947 | 0.522302381379847 | 7.03206435776325 |
| Firmicutes_Clostridia_Clostridiales_*Lachnospiraceae_Anaerostipes* | 0.0235141994988821 | 0.111158033994715 | 8.141789442452 |
| Firmicutes_Clostridia_Clostridiales_*Lachnospiraceae_Blautia* | 0.350037576931188 | 0.829908919180635 | 0.441950676144838 |
| Firmicutes_Clostridia_Clostridiales_*Lachnospiraceae_Coprococcus* | 0.63298792525428 | 0.910350471736992 | 1.61292856266408 |
| Firmicutes_Clostridia_Clostridiales_*Lachnospiraceae_Dorea* | 0.533188983845618 | 0.910350471736992 | 0.711338007447569 |
| Firmicutes_Clostridia_Clostridiales_*Lachnospiraceae_IncertaeSedis* | 0.647747730225295 | 0.910350471736992 | 0.702283214846625 |
| Firmicutes_Clostridia_Clostridiales_*Lachnospiraceae_Pseudobutyrivibrio* | 0.0052414560194734 | 0.0389365304303739 | 8.72960155944843 |
| Firmicutes_Clostridia_Clostridiales_*Lachnospiraceae_Roseburia* | 1.12126997671544e-05 | 0.000194353462630676 | 58.392370124539 |
| Firmicutes_Clostridia_Clostridiales_*Lachnospiraceae_uncultured* | 0.492086701190978 | 0.910350471736992 | 2.11261402931371 |
| Firmicutes_Clostridia_Clostridiales_*Peptostreptococcaceae_IncertaeSedis* | 0.875808732573474 | 0.948792793621264 | 1.21643480957773 |
| Firmicutes_Clostridia_Clostridiales_*Ruminococcaceae_Faecalibacterium* | 3.10570620906795e-06 | 8.07483614357668e-05 | 25.3775294397594 |
| Firmicutes_Clostridia_Clostridiales_*Ruminococcaceae_IncertaeSedis* | 0.421860064975225 | 0.907485713728266 | 0.401655906066979 |
| Firmicutes_Clostridia_Clostridiales_*Ruminococcaceae_Subdoligranulum* | 0.65045492691293 | 0.910350471736992 | 0.608671306992699 |
| Firmicutes_Clostridia_Clostridiales_*Ruminococcaceae_uncultured* | 0.000659196851342704 | 0.00685564725396412 | 64.6817282597586 |
| Firmicutes_Erysipelotrichia_Erysipelotrichales_*Erysipelotrichaceae_Catenibacterium* | 0.0257595596380067 | 0.111624758431362 | 68.5234950859945 |
| Firmicutes_Erysipelotrichia_Erysipelotrichales_*Erysipelotrichaceae_Solobacterium* | 0.0554245584894363 | 0.192138469430046 | 23.8152906008134 |
| Firmicutes_Negativicutes_Selenomonadales_*Acidaminococcaceae_Acidaminococcus* | 0.9724383781961 | 0.998455927930001 | 1.02746634776984 |
| Firmicutes_Negativicutes_Selenomonadales_*Acidaminococcaceae_uncultured* | 0.720795398000096 | 0.910350471736992 | 1.26563466077879 |
| Firmicutes_Negativicutes_Selenomonadales_*Veillonellaceae_Anaerospora* | 0.759562651916719 | 0.910350471736992 | 1.59631067821022 |

| | | | |
|---|---|---|---|
| Firmicutes_Negativicutes_Selenomonadales_*Veillonellaceae_Dialister* | 1.96278504817926e-11 | 1.02064822505322e-09 | 597.640570451125 |
| Firmicutes_Negativicutes_Selenomonadales_*Veillonellaceae_uncultured* | 0.511505543132624 | 0.910350471736992 | 2.26333477944056 |
| Firmicutes_Negativicutes_Selenomonadales_*Veillonellaceae_Veillonella* | 0.315150384225727 | 0.819390998986889 | 1.71921341839835 |
| Proteobacteria_Deltaproteobacteria_Desulfovibrionales_*Desulfovibrionaceae_Desulfovibrio* | 0.351115311961038 | 0.829908919180635 | 1.83855011686025 |
| Proteobacteria_Gammaproteobacteria_Enterobacteriales_*Enterobacteriaceae_Citrobacter* | 0.999445647555691 | 0.999445647555691 | 0.886398594755356 |
| Proteobacteria_Gammaproteobacteria_Enterobacteriales_*Enterobacteriaceae_Enterobacter* | 0.914014110774741 | 0.969974158373195 | 1.0405428991709 |
| Proteobacteria_Gammaproteobacteria_Enterobacteriales_*Enterobacteriaceae_Klebsiella* | 0.0483418011176831 | 0.179555261294251 | 5.05534662044924 |
| Proteobacteria_Gammaproteobacteria_Enterobacteriales_*Enterobacteriaceae_Proteus* | 8.90625467640891e-05 | 0.00115781310793316 | 34.9983942176548 |
| Proteobacteria_Gammaproteobacteria_Enterobacteriales_*Enterobacteriaceae_Salmonella* | 0.00211096167199588 | 0.0182950011572976 | 0.175488326262033 |
| Proteobacteria_Gammaproteobacteria_Enterobacteriales_*Enterobacteriaceae_Serratia* | 0.0101479575661776 | 0.0586326437156928 | 179.269747243992 |
| Proteobacteria_Gammaproteobacteria_Pasteurellales_*Pasteurellaceae_Haemophilus* | 0.0730037093686917 | 0.237262055448248 | 0.263140690902833 |
| Verrucomicrobia_Verrucomicrobiae_Verrucomicrobiales_*Verrucomicrobiaceae_Akkermansia* | 0.00662977324464491 | 0.0430935260901919 | 0.213023367084797 |
| **Taxon (Infection episode in the first 6 months)** | | | |
| Actinobacteria_Actinobacteria_Actinomycetales_*Actinomycetaceae_Actinomyces* | 0.647081047077153 | 0.854857109827581 | 1.18108591591995 |
| Actinobacteria_Actinobacteria_Bifidobacteriales_*Bifidobacteriaceae_Bifidobacterium* | 0.208700319384811 | 0.609780295419192 | 1.15661825379784 |
| Actinobacteria_Actinobacteria_Micrococcales_*Micrococcaceae_Rothia* | 0.480752896420976 | 0.806424213351315 | 0.828904093683049 |
| Actinobacteria_Coriobacteriia_Coriobacteriales_*Coriobacteriaceae_Collinsella* | 0.36113722819266 | 0.748521454706671 | 1.32786491210472 |
| Actinobacteria_Coriobacteriia_Coriobacteriales_*Coriobacteriaceae_Slackia* | 0.826074422245971 | 0.913954679931713 | 1.32914444406147 |
| Actinobacteria_Coriobacteriia_Coriobacteriales_*Coriobacteriaceae_uncultured* | 0.20840250929045 | 0.609780295419192 | 0.621858329254682 |

| | | | |
|---|---|---|---|
| Bacteroidetes_Bacteroidia_Bacteroidales_*Bacteroidaceae_Bacteroides* | 0.674021951979439 | 0.854857109827581 | 0.982692072362519 |
| Bacteroidetes_Bacteroidia_Bacteroidales_*Porphyromonadaceae_Barnesiella* | 0.732327924532027 | 0.906691716087272 | 0.860679477820878 |
| Bacteroidetes_Bacteroidia_Bacteroidales_*Porphyromonadaceae_Macellibacteroides* | 0.303260679646402 | 0.748521454706671 | 0.584647467141178 |
| Bacteroidetes_Bacteroidia_Bacteroidales_*Porphyromonadaceae_Paludibacter* | 0.996926598041377 | 0.996926598041377 | 1.00151105178645 |
| Bacteroidetes_Bacteroidia_Bacteroidales_*Rikenellaceae_Alistipes* | 0.58206751321655 | 0.846976173279335 | 0.658632934541159 |
| Firmicutes_Bacilli_Bacillales_*Bacillaceae_Bacillus* | 0.766654199689884 | 0.913954679931713 | 0.901580270002105 |
| Firmicutes_Bacilli_Bacillales_*FamilyXIIncertaeSedis_Gemella* | 0.79160555697773 | 0.913954679931713 | 0.940806608559011 |
| Firmicutes_Bacilli_Bacillales_*Staphylococcaceae_Staphylococcus* | 0.425143176775629 | 0.762325696287335 | 1.16466615162787 |
| Firmicutes_Bacilli_Lactobacillales_*Carnobacteriaceae_Granulicatella* | 0.794686644942205 | 0.913954679931713 | 0.914722558925883 |
| Firmicutes_Bacilli_Lactobacillales_*Enterococcaceae_Enterococcus* | 0.211077794568182 | 0.609780295419192 | 1.56102017753302 |
| Firmicutes_Bacilli_Lactobacillales_*Lactobacillaceae_Lactobacillus* | 0.505149780373465 | 0.82086839310688 | 0.794181005616416 |
| Firmicutes_Bacilli_Lactobacillales_*Streptococcaceae_Streptococcus* | 0.82297518716924 | 0.913954679931713 | 1.04039095331817 |
| Firmicutes_Clostridia_Clostridiales_*Clostridiaceae_Clostridium* | 0.422855131179287 | 0.762325696287335 | 0.780082367225292 |
| Firmicutes_Clostridia_Clostridiales_*FamilyXIIncertaeSedis_Finegoldia* | 0.553691375839063 | 0.846976173279335 | 1.35780579984363 |
| Firmicutes_Clostridia_Clostridiales_*FamilyXIIncertaeSedis_Parvimonas* | 0.643816948903431 | 0.854857109827581 | 1.28660499893556 |
| Firmicutes_Clostridia_Clostridiales_*FamilyXIIncertaeSedis_Peptoniphilus* | 3.7221261604941e-05 | 0.000387101120691386 | 8.60674774993136 |
| Firmicutes_Clostridia_Clostridiales_*Lachnospiraceae_Anaerostipes* | 6.06372409584189e-08 | 1.57656826491889e-06 | 0.0807906502373632 |
| Firmicutes_Clostridia_Clostridiales_*Lachnospiraceae_Blautia* | 0.587519052647836 | 0.846976173279335 | 0.815129687652207 |
| Firmicutes_Clostridia_Clostridiales_*Lachnospiraceae_Coprococcus* | 0.0549451862256457 | 0.317461075970397 | 2.17588920046295 |
| Firmicutes_Clostridia_Clostridiales_*Lachnospiraceae_Dorea* | 0.380467485537476 | 0.748521454706671 | 1.77866580789854 |
| Firmicutes_Clostridia_Clostridiales_*Lachnospiraceae_IncertaeSedis* | 0.204965968955269 | 0.609780295419192 | 1.5459396262432 |

| | | | |
|---|---|---|---|
| Firmicutes_Clostridia_Clostridiales_*Lachnospiraceae_Pseudobutyrivibrio* | 3.36499771633692e-05 | 0.000387101120691386 | 3.06367421221352 |
| Firmicutes_Clostridia_Clostridiales_*Lachnospiraceae_Roseburia* | 0.000451596384909735 | 0.0039138353358843 7 | 4.38779821019686 |
| Firmicutes_Clostridia_Clostridiales_*Lachnospiraceae_uncultured* | 0.124898622371034 | 0.463909168806696 | 1.92782721952416 |
| Firmicutes_Clostridia_Clostridiales_*Peptostreptococcaceae_IncertaeSedis* | 0.103394995670941 | 0.413579982683764 | 0.372095238890143 |
| Firmicutes_Clostridia_Clostridiales_*Ruminococcaceae_Faecalibacterium* | 1.40941459138631e-17 | 7.32895587520882e-16 | 11.0580243725353 |
| Firmicutes_Clostridia_Clostridiales_*Ruminococcaceae_IncertaeSedis* | 0.579440437473355 | 0.846976173279335 | 0.981679058450112 |
| Firmicutes_Clostridia_Clostridiales_*Ruminococcaceae_Subdoligranulum* | 0.602656123294911 | 0.846976173279335 | 0.803286460661226 |
| Firmicutes_Clostridia_Clostridiales_*Ruminococcaceae_uncultured* | 0.35751716168729 | 0.748521454706671 | 1.63455876782395 |
| Firmicutes_Erysipelotrichia_Erysipelotrichales_*Erysipelotrichaceae_Catenibacterium* | 0.460240522958423 | 0.7977502397946 | 0.812627207721459 |
| Firmicutes_Erysipelotrichia_Erysipelotrichales_*Erysipelotrichaceae_Solobacterium* | 0.387158671921288 | 0.748521454706671 | 0.514884221815122 |
| Firmicutes_Negativicutes_Selenomonadales_*Acidaminococcaceae_Acidaminococcus* | 0.0743219642222087 | 0.351340194504986 | 3.43008872937745 |
| Firmicutes_Negativicutes_Selenomonadales_*Acidaminococcaceae_uncultured* | 0.0991032489607555 | 0.413579982683764 | 3.01372508937399 |
| Firmicutes_Negativicutes_Selenomonadales_*Veillonellaceae_Anaerospora* | 0.00568047557982504 | 0.042197818592986 | 7.50831096639833 |
| Firmicutes_Negativicutes_Selenomonadales_*Veillonellaceae_Dialister* | 0.852745973007942 | 0.920593092775817 | 1.2129884804765 |
| Firmicutes_Negativicutes_Selenomonadales_*Veillonellaceae_uncultured* | 0.885185666130593 | 0.920593092775817 | 0.91518275301533 |
| Firmicutes_Negativicutes_Selenomonadales_*Veillonellaceae_Veillonella* | 0.0122119580036156 | 0.0793777270235012 | 0.914207874189783 |
| Proteobacteria_Deltaproteobacteria_Desulfovibrionales_*Desulfovibrionaceae_Desulfovibrio* | 0.325176695373587 | 0.748521454706671 | 2.13380778023727 |
| Proteobacteria_Gammaproteobacteria_Enterobacteriales_*Enterobacteriaceae_Citrobacter* | 0.388655370713079 | 0.748521454706671 | 0.80214886645561 |
| Proteobacteria_Gammaproteobacteria_Enterobacteriales_*Enterobacteriaceae_Enterobacter* | 0.882778351241202 | 0.920593092775817 | 0.974481740506987 |

| | | | |
|---|---|---|---|
| Proteobacteria_Gammaproteobacteria_Enterobacteriales_*Enterobacteriaceae_Klebsiella* | 0.918698334954752 | 0.936712027797002 | 1.03536327285497 |
| Proteobacteria_Gammaproteobacteria_Enterobacteriales_*Enterobacteriaceae_Proteus* | 2.88071691986787e-07 | 4.9932426611043e-06 | 12.8287754287656 |
| Proteobacteria_Gammaproteobacteria_Enterobacteriales_*Enterobacteriaceae_Salmonella* | 0.665612331315147 | 0.854857109827581 | 0.874151322304419 |
| Proteobacteria_Gammaproteobacteria_Enterobacteriales_*Enterobacteriaceae_Serratia* | 0.357845814123735 | 0.748521454706671 | 0.766877425316356 |
| Proteobacteria_Gammaproteobacteria_Pasteurellales_*Pasteurellaceae_Haemophilus* | 0.232392524214889 | 0.636021645219697 | 0.681148442030422 |
| Verrucomicrobia_Verrucomicrobiae_Verrucomicrobiales_*Verrucomicrobiaceae_Akkermansia* | 0.0618481388774566 | 0.321610322162775 | 0.366217766481984 |

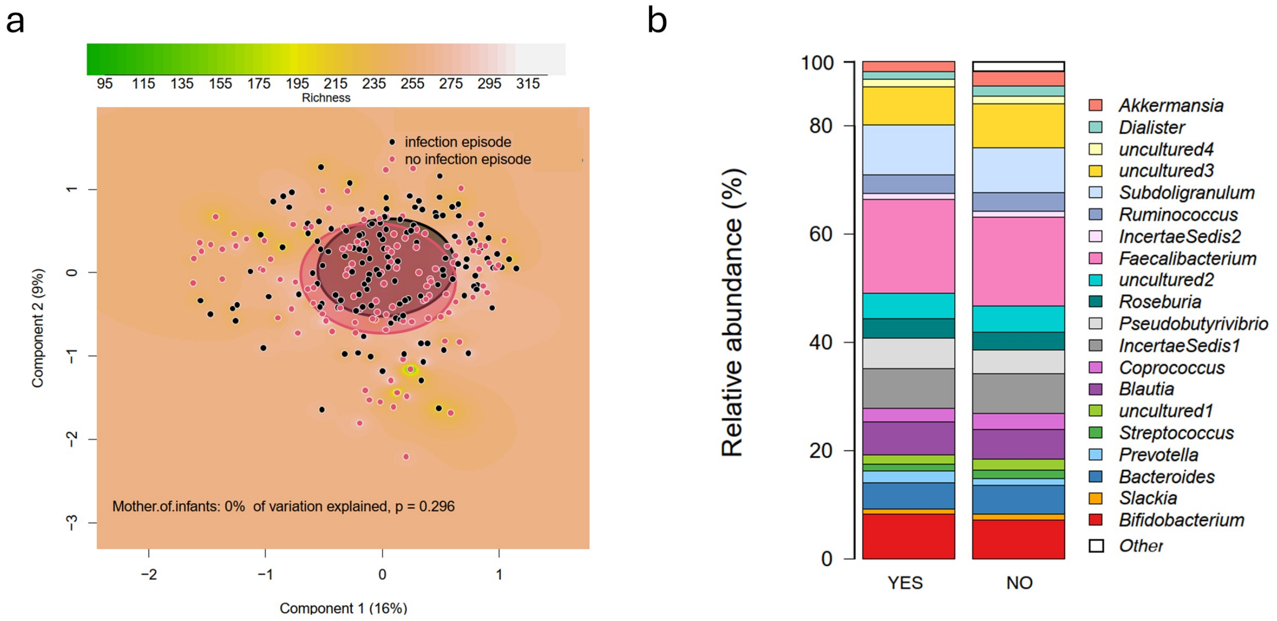


**Supplemental Figure S1. Principal coordinate analysis (PCoA) and relative abundances of the maternal microbiota at genus level. Comparison between mothers of infants who developed a respiratory tract infection episode in the first six months of age and mothers of randomly selected infants with no such infection episode in the first six months of age.** PCoA plots based on Bray-Curtis dissimilarities of the samples, showing as background richness of the microbiota (a). Clusters are shown by circles, which were drawn based on the standard deviations of the data points in each category of the samples. The comparisons were between mothers of infants who developed a respiratory tract infection in the first six months of age and mothers of infants remaining healthy (p=0.3). Clustered stacked column graphs demonstrate microbiota differences at the genus level (b).

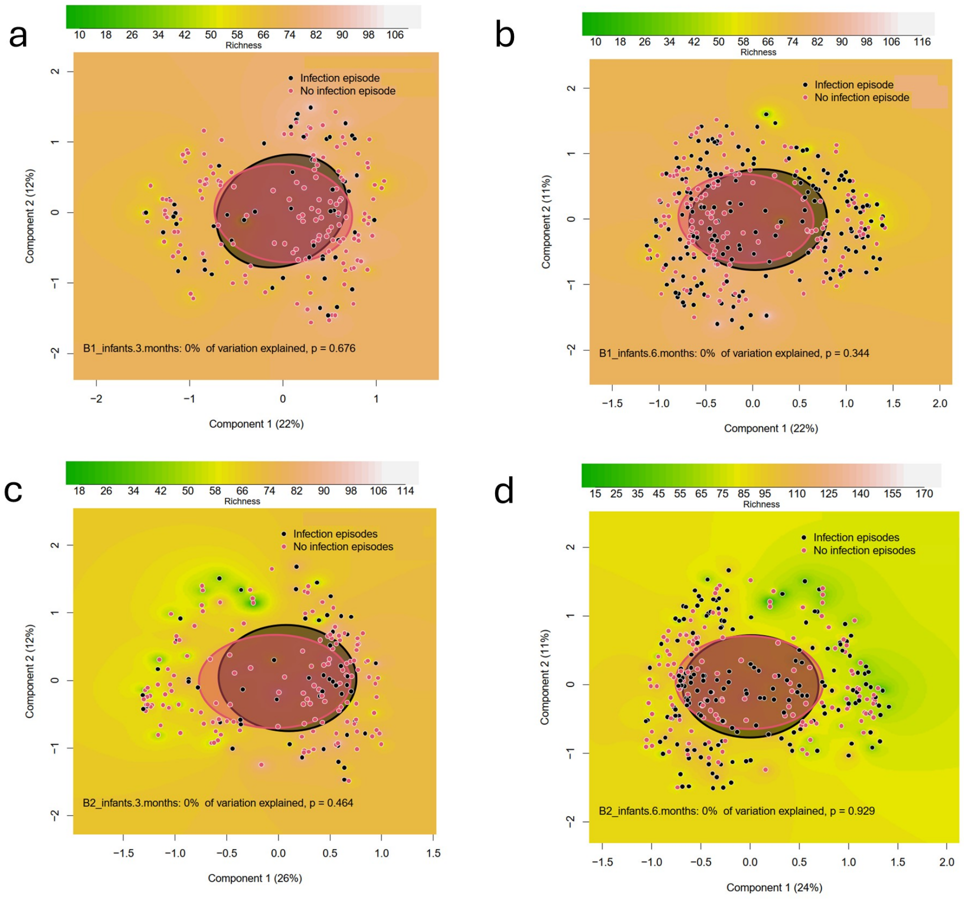


**Supplemental Figure S2. Principal coordinate analysis (PCoA) of the infant microbiota at three and six weeks of age between infants who developed a respiratory tract infection episode in the first three and six months of life and infants in the control group at genus level.** PCoA plots are based on Bray-Curtis dissimilarities of the samples, showing as background richness of the microbiota at time points of three and six weeks of age. Clusters are shown by circles, which were drawn based on the standard deviations of the data points in each category of the samples. At week three, the comparisons were between infants who developed a respiratory tract infection episode in the first three (a, p=0.68) and six (b, p=0.34) months of life and randomly selected infants remaining healthy in the first six months of life. At week six the comparisons were between infants who developed a respiratory tract infection episode in the first three (c, p=0.46) and six (d, p=0.93) months of life and randomly selected infants remaining healthy in the first six months of life.

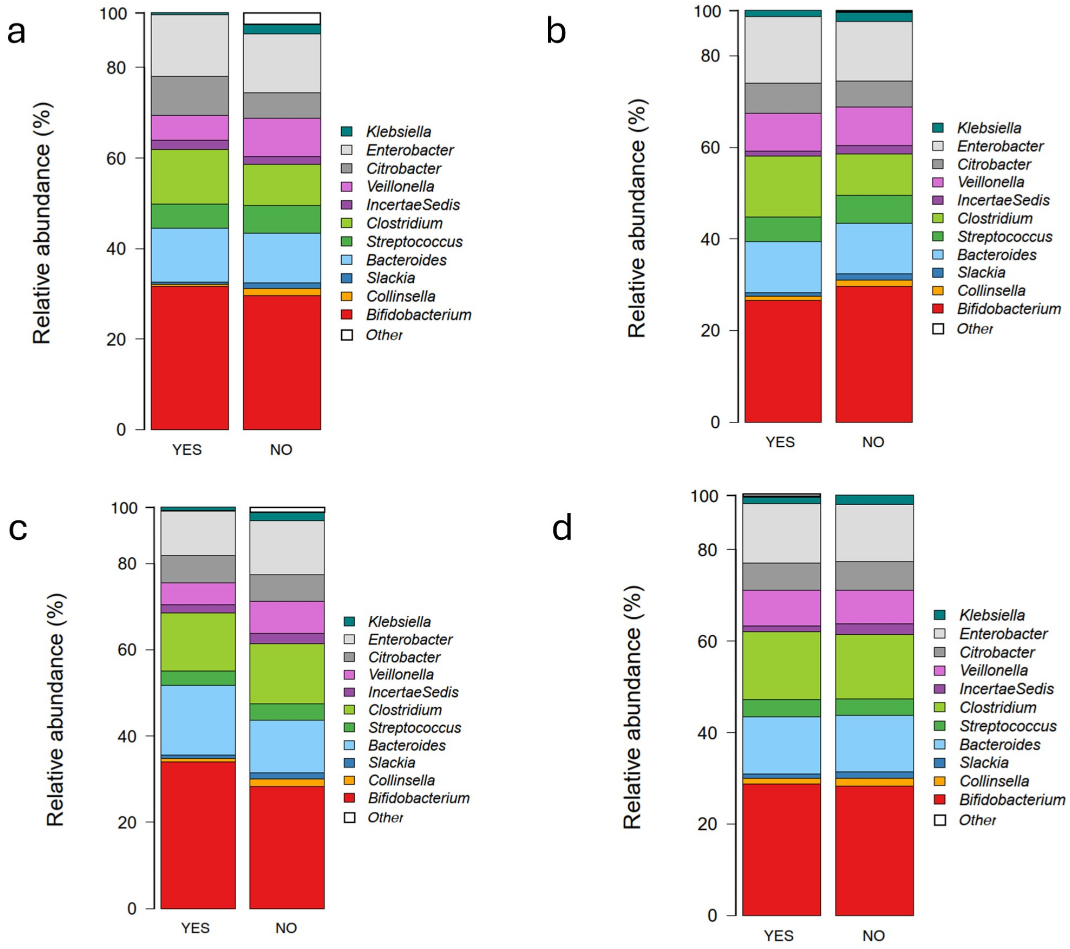


**Supplemental Figure S3. Relative abundances of the infant microbiota at three and six weeks of age between infants who developed a respiratory tract infection episode in the first three and six months of life and infants in the control group at genus level.** Clustered stacked column graphs demonstrate microbiota differences at genus level at time points of three and six weeks of age. At week three, the comparisons were between infants who developed a respiratory tract infection episode (YES) in the first three (a) and six (b) months of life and randomly selected infants remaining healthy (NO) in the first six months of life. At week six the comparisons were between infants who developed a respiratory tract infection episode (YES) in the first three (c) and six (d) months of life and randomly selected infants remaining healthy (NO) in the first six months of life.

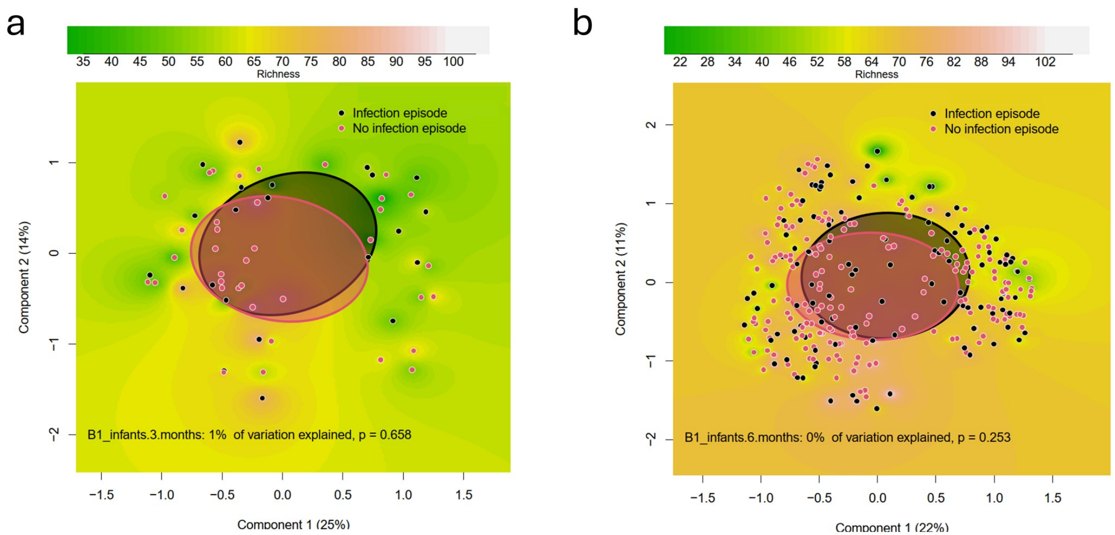


**Supplemental Figure S4. Principal coordinate analysis (PCoA) of the sensitivity analysis. Infant microbiota at three weeks of age between infants who developed a respiratory tract infection episode in the first three and six months of age and matched infants in the control group at genus level.** PCoA plots are based on Bray-Curtis dissimilarities of the samples, showing as background richness of the microbiota. Clusters are shown by circles, which were drawn based on the standard deviations of the data points in each category of the samples. The comparisons are between infants who developed a respiratory tract infection episode in the first three (a, p=0.66) and six (b, p= 0.25) months of life compared to matched infants with no such infection episodes in the first three and six months of life. Infants with any infections before the 3-week stool sample was taken were excluded.

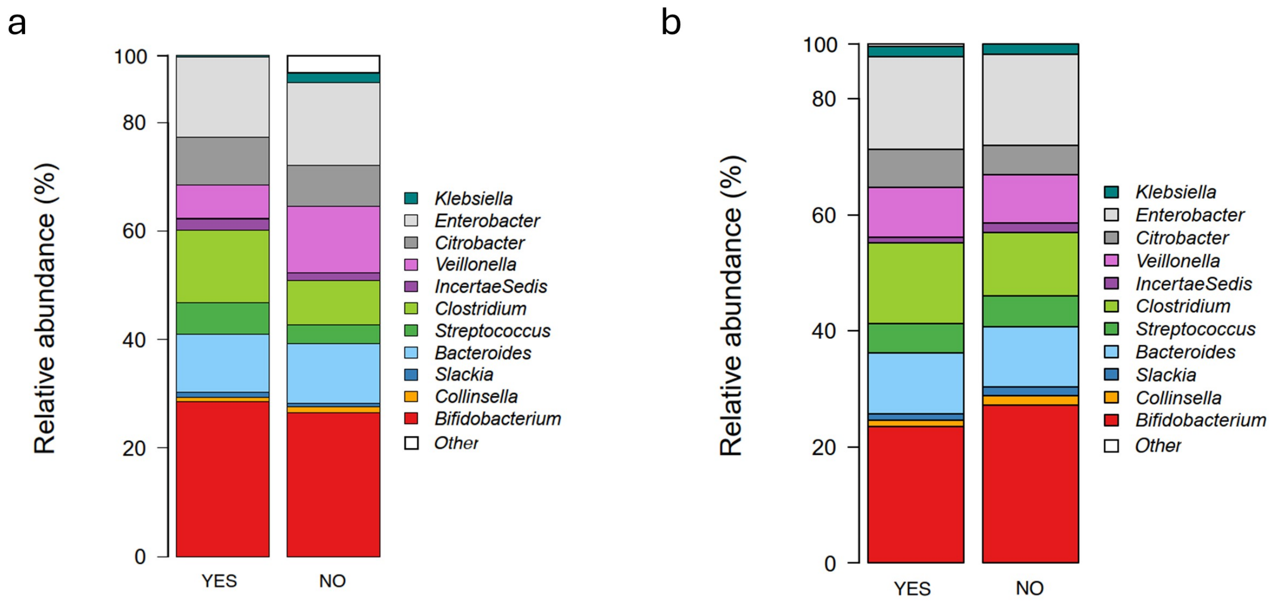


**Supplemental Figure S5. Relative abundances of the major taxa of the sensitivity analysis at genus level. Infant microbiota at three weeks of age between infants who developed a respiratory tract infection episode in the first three and six months of life and matched infants in the control group.** The Clustered stacked column graphs demonstrate microbiota differences at genus level at three weeks of age. The comparisons are between infants who developed a respiratory tract infection episode (YES) in the first three (a) and six (b) months of life and carefully selected matched infants remaining healthy (NO) in the first six months of life. Infants with any infections before the 3-week stool sample was taken were excluded.
